# Optimization of Compost and Peat Mixture Ratios for Production of Pepper Seedlings

**DOI:** 10.3390/ijms26020442

**Published:** 2025-01-07

**Authors:** Anita Zapałowska, Wacław Jarecki, Andrzej Skwiercz, Tadeusz Malewski

**Affiliations:** 1 Department of Agriculture and Waste Management, University of Rzeszów, St. Ćwiklinskiej 1a, 35-601 Rzeszów, Poland; 2 Department of Crop Production, University of Rzeszów, St. Zelwerowicza 4, 35-601 Rzeszów, Poland; wjarecki@ur.edu.pl; 3 Department of Plant Protection, The National Institute of Horticultural Research, Konstytucji 3 Maja 1/3, 96-100 Skierniewice, Poland; andrzej.skwiercz@inhort.pl; 4 Department of Molecular and Biometric Techniques, Museum and Institute of Zoology, Polish Academy of Sciences, 00-818 Warsaw, Poland; tmalewski@miiz.waw.pl

**Keywords:** horticultural substrates, chlorophyll content, plant biometrics, nematodes, *Capsicum annuum*

## Abstract

Substituting peat moss with compost derived from organic waste in plant nurseries presents a promising solution for reducing environmental impact, improving waste management, and enhancing soil health while promoting sustainable agricultural practices. However, selecting the appropriate proportions of both materials is crucial for each plant species. This study investigates the effects of different ratios of compost and peat mixtures on the growth and development of pepper seedlings. The compost mixtures used in the study included the following combinations: sewage sludge with sawdust (A), sewage sludge with sawdust and biodegradable garden/park waste (B), and biodegradable garden/park waste with sawdust (C). The final substrates used for seedling production were composed of composts (A, B, C) and peat (O) as a structural additive, mixed in different proportions by mass: I-O 25%, II-O 50%, and III-O 75%. Seedlings grown in these substrates were assessed using biometric and physiological measurements. Nematode species present in substrates were identified by metabarcoding analysis. The results revealed that substrate productivity depended not only on nutrient content but also on structural properties, which were significantly influenced by the peat proportion. Among the tested compost mixtures, variant A I emerged as the most effective substrate, promoting optimal seedling growth. Molecular nematode analysis revealed significant nematode contamination in substrates with higher peat proportions (C II and C III), including *Meloidogyne* sp. *Lichtenburg* (26%), *Meloidogyne hispanica* (5%), *Meloidogyne* sp. *Mi_c1* (3%), *Meloidogyne ethiopica* (2%), and *Meloidogyne thailandica* (1%). The findings underscore the critical importance of achieving an optimal balance between nutrient content and structural properties in substrates to support the healthy growth and development of pepper seedlings. To further enhance crop performance and reduce the risk of pest-related damage, it is essential to prioritize the improvement of substrate selection strategies. Monitoring for nematode contamination is crucial to prevent potential compromises in seedling quality and overall productivity.

## 1. Introduction

Pepper (*Capsicum* spp.) ranks among the most significant vegetable crops globally in terms of production. Cultivated across the world, peppers are valued for their remarkable diversity in fruit shape, size, and color [1,2]. Pepper production in Poland has remained stable over recent years, with 2023 data from the Agency for Restructuring and Modernisation of Agriculture (ARMA) showing cultivation across 2585 hectares. Of this, slightly more than 600 hectares are dedicated to tunnel cultivation. However, the widespread reliance on monoculture, coupled with specific climatic and cultivation conditions, has introduced several agrotechnical challenges. One of the primary issues is yield reduction, largely attributed to monoculture practices and the excessive use of mineral fertilizers. These practices contribute to soil salinization and hinder nutrient absorption, negatively affecting plant health and productivity. As a thermophilic crop, pepper is particularly sensitive to weather fluctuations, making consistent and careful monitoring throughout the production process essential for achieving successful cultivation outcomes.

In response to these challenges, the importance of soilless cultivation is steadily increasing worldwide [3,4]. This method offers a sustainable approach to plant production. The selection of a suitable substrate plays a crucial role in the successful cultivation of seedlings [5]. Effective substrates should be rich in plant nutrients, provide adequate water retention and aeration, and remain cost-effective to ensure practicality and efficiency [6,7].

With peat accounting for approximately 23% of the total cost of seedling production [8], there is a growing need to identify cost-effective and sustainable alternatives. Even though peat extraction sites are typically marginal patches within broader landscapes, they can be key sites for biogeochemical flows of carbon and nutrients and might be key sites for restoring flora and fauna.

One promising substitute is vermicompost, which has gained considerable attention for its use in vegetable cultivation [9,10]. Unlike peat, vermicompost is derived from renewable resources, making it an environmentally friendly and sustainable material [11].

The use of compost provides an opportunity to replace energy-intensive substrates such as rockwool or perlite [12]. Acting as a slow-release fertilizer, compost helps conserve nutrients like nitrogen, which are costly and energy-demanding to produce. With the price of synthetic nitrogen nearly tripling over the past decade, many growers are increasingly turning to compost as a cost-effective alternative for a steady supply of nitrogen and other essential nutrients [13]. However, some organic substrates may be deficient in nutrients, in which case supplementing with nitrogen fertilizers may be a practical option to ensure optimal plant growth. Nitrogen fertilizers are energy-intensive to produce and rely heavily on natural gas in the Haber–Bosch process. Rising energy prices increase their costs, burdening farmers, especially in developing regions, and making agriculture less profitable. High costs often lead to reduced fertilizer use, potentially leading to lower yields and threatening food security, especially in regions dependent on high-input agriculture. Recycling compost-based media offers an environmentally friendly solution for the safe disposal or reuse of substrates at the end of their life cycle. These used substrates can be reused as soil amendments or integrated into growing media for less demanding crops, supporting a more sustainable and circular agricultural production system. By replacing energy-intensive substrates and synthetic fertilizers with compost, global agriculture can transition to more resilient, sustainable, and economically viable practices, benefiting farmers, consumers, and the environment [14,15,16].

A variety of abiotic and biotic factors influence the establishment and composition of nematode communities in compost and soil. In agricultural systems, the proportion and diversity of nematodes serve as valuable indicators for assessing soil process rates [17] and overall soil functions [18]. Compost, with its rich organic matter content, can significantly impact nematode populations by providing a habitat that supports both free-living beneficial nematodes [19] and, in some cases, plant-parasitic species [20]. Renčo et Kováčik [21] have found the suppressive effects of various doses of vermicomposts produced from municipal wastes on the soil populations of two species of potato-cyst nematodes: *G. rostochiensis* and *G. pallida*. Understanding these interactions is crucial for managing soil health and optimizing compost use in sustainable agriculture.

The market offers a wide range of commercial substrates for vegetable cultivation, yet growing pressure for sustainability in farming has prompted the exploration of alternative, more eco-friendly options, such as the use of compost. When available, compost serves as a valuable source of organic matter, particularly as a sustainable substitute for synthetic fertilizers. However, a significant knowledge gap remains regarding the optimal use of compost–peat mixtures for pepper cultivation. While compost is increasingly recognized for its sustainability, the specific effects of different compost-to-peat ratios on pepper seedling growth are still underexplored.

This study addresses that gap by examining how various compost–peat combinations influence seedling development, offering critical insights into the role of nutrient content, structural properties, and nematode contamination. The study’s focus on nematode contamination provides valuable insights for developing pest management strategies, which are essential for sustaining healthy crops across diverse agricultural systems.

We hypothesized that that compost–peat mixtures could serve as effective and sustainable alternatives for pepper seedling cultivation, enhancing growth performance while mitigating the risk of nematode infestations.

## 2. Results and Discussion

### 2.1. Chemical Properties of the Substrates

Nitrate (N), phosphorus (P), potassium (K), magnesium (Mg), and calcium (Ca) content varied according to the compost type.

Variant A contained the highest nitrogen (N) levels, with an average of 40 g/kg. The average contents of available forms of phosphorus (P) and potassium (K) were 13.0 and 4.9 g/kg, while the overall contents of magnesium (Mg) and calcium (Ca) were 3.2 and 17.1 g/kg. The determined pH value of the variant A was 5.9 and the C:N ratio was 19.25.

Variant B had an average nitrogen (N) content of 18.2 g/kg. The available forms of phosphorus (P) and potassium (K) averaged 10 g/kg and 6.88 g/kg, respectively. The total concentrations of magnesium (Mg) and calcium (Ca) were 3.2 g/kg and 15.2 g/kg. The pH value of variant B was 6.3, and its C:N ratio was 23.57.

**Figure 1:**
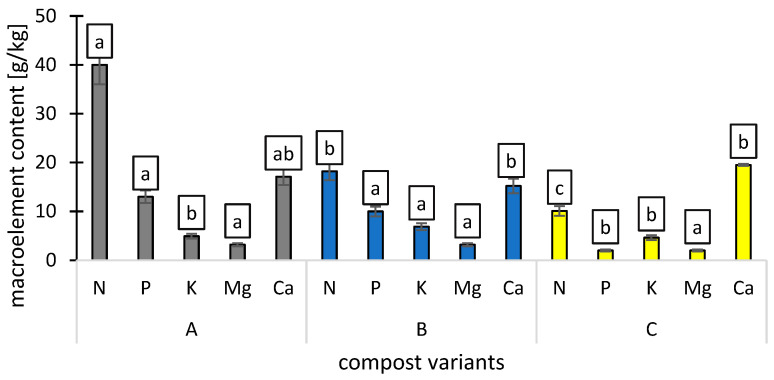
Macroelement (N, P, K, Mg, and Ca) contents [g/kg] of compost variants (A, B, C), n = 3. Different letters in the columns indicate significant differences (*p* < 0.05). The standard error is marked on the columns.

Variant C exhibited an average nitrogen (N) content of 10 g/kg. The concentrations of available phosphorus (P) and potassium (K) were 2 g/kg and 4.6 g/kg, respectively. The total magnesium (Mg) and calcium (Ca) levels were 2 g/kg and 9.5 g/kg. The pH of variant C was measured at 6.3, with a C:N ratio of 37.72 (Figure 1).

The average contents of available forms of phosphorus (P) and potassium (K) in deacidified peat (O) were 0.03 and 0.21 g/kg, while the overall contents of magnesium (Mg) and calcium (Ca) were 0.09 and 0.84 g/kg. Available nitrates (N) were 0.24 g/kg, C org 44.1%, and pH at 5.6.

Nitrogen (N) is essential for plant growth, being a key component of proteins and chlorophyll. The higher nitrogen levels in Variant A (40 g/kg) are attributed to a greater concentration of nitrogen-rich materials [22]. The lower nitrogen in Variants B (18.2 g/kg) and C (10 g/kg) suggests potential nitrogen loss during composting, possibly due to microbial degradation or the C:N ratio, which affects decomposition [23]. Variant A had the highest phosphorus (13.0 g/kg) and potassium (4.9 g/kg) contents, essential for energy transfer and root development [24]. Magnesium and calcium levels varied among the variants, with Variant A containing the highest calcium concentration (17.1 g/kg). The pH range of 5.5 to 7.0 is generally considered optimal for most vegetable crops [25]. The pH values of 5.9 to 6.3 observed in this study were within this range, suggesting favorable conditions for nutrient availability.

Pepper grows best in light-textured soils that provide good drainage and sufficient water-holding capacity. The ideal pH range is 5.5 to 7.0, with acidic soils requiring liming. At low pH levels, plants struggle to absorb nitrogen as well as other essential nutrients. The pH and compost type shape the chemical, biological, and physical environment of the root zone, influencing nutrient uptake, plant health, stress resistance, and ultimately the quality and yield of pepper crops. These factors integrate into a complex system that supports optimal and resilient cultivation.

**Figure 2:**
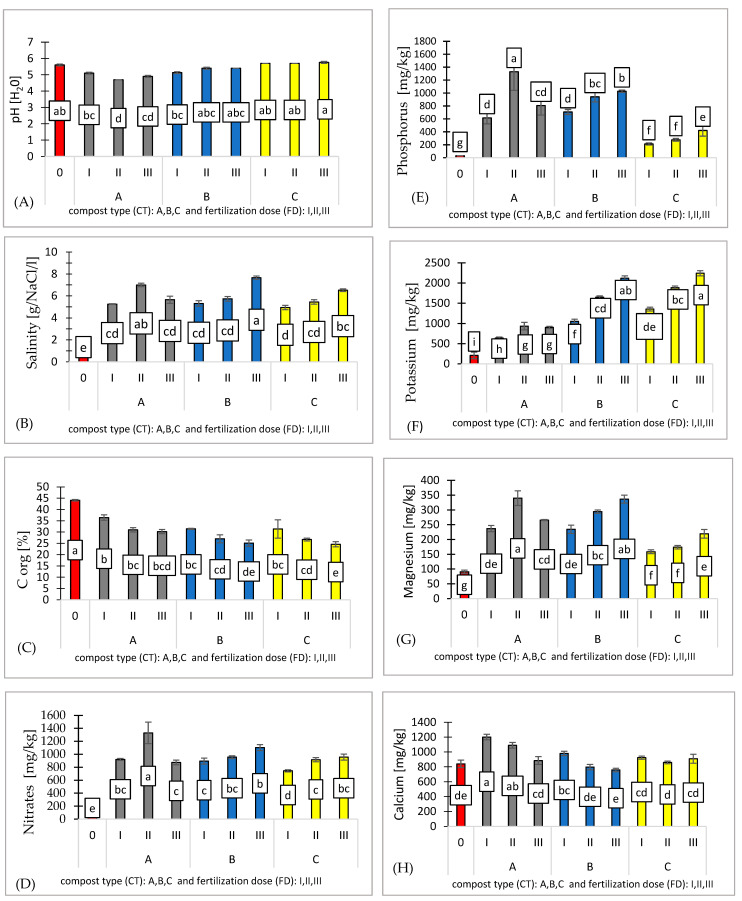
Effect of compost type (CT) and fertilization dose (FD) on substrates (0, AI, AII, AIII, BI, BII, BIII, CI, CII, CIII): pH [H_2_O] (**A**), salinity [g/NaCl/l] (**B**), Corg [%] (**C**), nitrates [mg/kg] (**D**), phosphorus [mg/kg] (**E**), potassium [mg/kg] (**F**), magnesium [mg/kg] (**G**), and calcium [mg/kg] (**H**). n = 3. Note: LSD for α = 0.05 for the impact of compost type (CT), fertilization dose (FD), and the interaction between tested parameters (CT)/(FD). Different letters in the columns indicate significant differences (*p* < 0.05). The standard error is marked on the columns.

The substrates used in experiment demonstrated a range of physicochemical properties based on the compost type. The compost treatments affected the substrate characteristics, with pH values ranging from 4.70 to 5.80, the lowest being recorded in substrate A (II) and the highest in substrate C (III) (Figure 2A).

The compost variants significantly affected the salinity levels, which ranged from 4.9 to 7.66. Substrate C (I) exhibited the lowest salinity, while substrate B (III) showed the highest salinity values (Figure 2B). Salinity is defined as one of the critical factors limiting the productivity of agricultural crops due to its negative impact on germination, plant vigor, plant growth, development, and crop yield.

Fertilization dose significantly impacts organic carbon content, and all treatments resulted in lower carbon content compared to the control (Corg. 44.1%) (Figure 2C).

When organic matter is added to the soil, it must undergo decomposition to release nutrients that can be absorbed by plants. The decomposition process occurs through two main pathways: a faster bacterial-driven channel and a slower fungal-based channel. The predominant decomposition pathway is influenced by factors such as soil ecosystem types and nutrient ratios, including the carbon to nitrogen (C:N) ratio. Additionally, bacteria and fungi, as the primary decomposers in the soil food web, can also immobilize inorganic nutrients, making them unavailable for plant uptake.

The carbon to nitrogen (C:N) ratio plays a significant role in shaping the soil ecosystem, including the population dynamics of nematodes. A high C:N ratio, often associated with plant residues or organic matter rich in carbon, can result in slower decomposition, as microorganisms focus on breaking down carbon-rich material. This slower decomposition can limit the availability of nitrogen, affecting the abundance and activity of nitrogen-cycling organisms like nematodes. In contrast, a lower C:N ratio, where nitrogen is more readily available, can lead to faster decomposition, which may increase nitrogen availability in the soil. This, in turn, can promote higher nematode populations, particularly those that feed on bacteria and fungi involved in nutrient cycling. Nematodes, especially bacterivorous and fungivorous types, play a crucial role in mineralizing nitrogen and other nutrients, thus contributing to soil fertility.

Overall, the C:N ratio influences the rate of decomposition, nutrient availability, and, consequently, the nematode populations that contribute to soil nutrient cycling.

Nematodes play an important role in the essential process of soil organic matter decomposition.

The fertilizer requirements for pepper, ranging from 100 to 170 kg/ha of nitrogen (N), are crucial for optimal growth. The high nitrate (N) concentrations found in substrate A (1330 mg/kg) suggest that this compost type is particularly effective at enhancing nitrate availability in the substrate, making it a valuable resource for pepper cultivation. In contrast, the significantly lower nitrate levels observed in substrate C (745 mg/kg) indicate that compost C does not provide the same level of nutrient availability. This difference in nutrient release is likely due to the distinct composition of the composts, with substrate A offering a more favorable nutrient profile for pepper seedlings (Figure 2D).

The significant role of nematodes in nitrogen mineralization, especially free-living nematodes that contribute to soil nutrient cycling by excreting soluble nitrogen, supports the nutrient dynamics within the substrates. This process enhances nitrogen availability, which is crucial for promoting plant growth [19].

The combined effect of compost type and fertilization dose has a significant impact on phosphorus (P) availability in the substrate, as reflected by the LSD values. The compost applications modified the substrate properties, resulting in available phosphorus levels ranging from 213 to 1330 mg/kg, with the lowest levels observed in substrate C and the highest in substrate A (Figure 2E).

It was observed that higher fertilization doses (I, II, III) have a positive impact on potassium (K) availability, with increasing doses leading to progressively higher potassium concentrations in the substrate. Compost C significantly enhances potassium availability, with the highest concentrations (2241 mg/kg) observed across all fertilization doses. Compost A provides the lowest potassium concentrations (638 mg/kg), though it still shows an increase with higher fertilization doses (Figure 2F).

A similar trend is observed, as the compost variants influenced substrate characteristics, with available magnesium (Mg) concentrations ranging from 158.6 to 340 mg/kg. The lowest levels were observed in substrate C and the highest in substrate A (Figure 2G).

Calcium is essential for maintaining cell wall structure, while magnesium (Mg) plays a central role in chlorophyll production and is crucial for photosynthesis [26,27,28].

The observed differences in available calcium (Ca) concentrations across the compost variants can be attributed to the distinct compositions and nutrient release characteristics of each compost type. Compost A, which showed the highest calcium levels (1201 mg/kg) likely contains compounds that facilitate its release into the substrate. On the other hand, compost B, which recorded the lowest calcium levels (758 mg/kg), may have a composition which limits its capacity to increase the available calcium in the substrate (Figure 2H).

The Pearson correlation coefficients indicated strong positive correlations between salinity and nitrates (N-NO_3_) (r = 0.915), phosphorus (P) and nitrates (N-NO_3_) (r = 0.815), and magnesium (Mg) and nitrates (N-NO_3)_ (r = 0.872). These results suggest that higher salinity, phosphorus (P), and magnesium (Mg) levels are associated with increased nitrates (N-NO_3_) concentrations, reflecting a potential relationship between these variables in the system. Conversely, negative correlations were observed for pH and phosphorus (P) (r = −0.724), potassium (K) and organic carbon (Corg) (r = −0.888), and magnesium (Mg) and pH (r = −0.619). These negative values indicate that as pH increases, phosphorus and magnesium levels decrease, while higher potassium levels are associated with reduced organic carbon (Corg). Such findings highlight the inverse relationships between these parameters, which may reflect underlying chemical or biological processes within the environment.

### 2.2. Seedling Growth Characteristics

**Figure 3:**
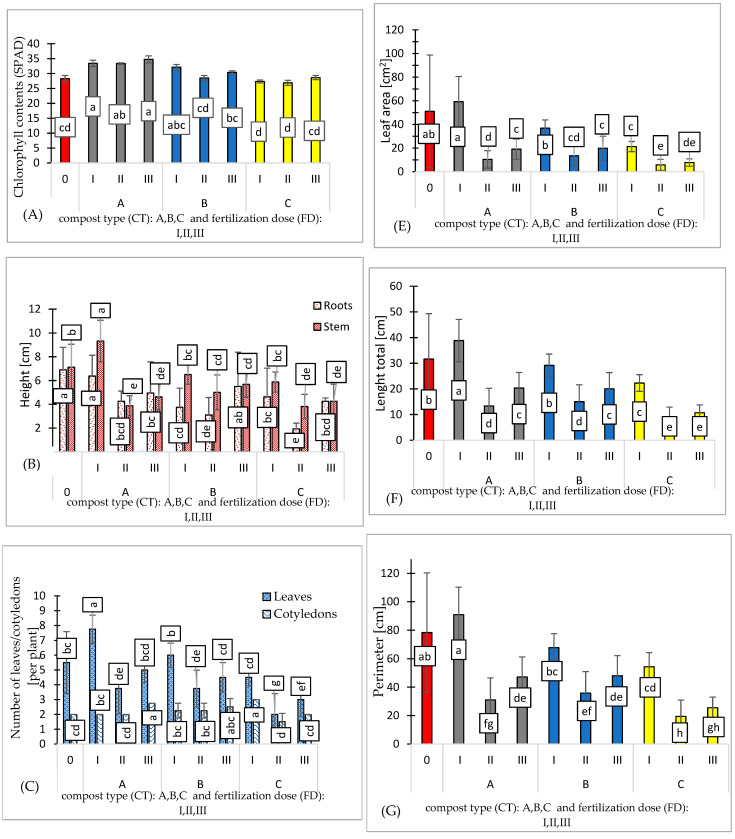

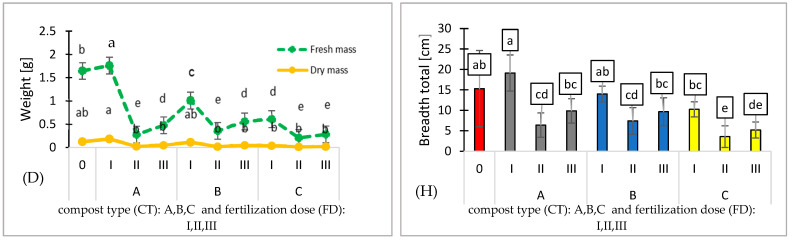
Effect of compost type (CT) and fertilization dose (FD) on chlorophyll contents [SPAD] (**A**), seedling height [cm] (**B**), number of leaves/cotyledons [per plant] (**C**), wet/dry weight [g] (**D**), leaf area [cm^2^] (**E**), length total [cm] (**F**), perimeter [cm] (**G**), and breadth total (cm) (**H**). N = 3. Note: LSD for α = 0.05 for the impact of compost type (CT), fertilization dose (FD), and the interaction between tested parameters (CT)/(FD). Different letters in the columns indicate significant differences (*p* < 0.05). The standard error is marked on the columns.

The seed material of the Marta Polka variety was certified and characterized by high germination capacity. Chlorophyll content (SPAD) ranged from 26.87 to 34.87 value, with the lowest observed in variant C and the highest in substrate A (Figure 3A).

The height of pepper seedlings ranged from 1.95 to 6.37 cm for the roots, with the lowest observed in variant C (II) and the highest in substrate A (I). For the stem, the highest was observed in variant A (I), at 9.32, while the lowest was in variants A (II) and C (II), at 3.81 cm (Figure 3B).

The number of leaves per plant ranged from 2 to 7.75 in variant C (II) and variant A (I), respectively. (Figure 3C). Proper growth and nutrition of seedlings are critical after transplantation to their final location. The use of high-quality, healthy, and vigorous seedlings is essential, as their absence can significantly impair the achievement of optimal yield potential.

High soil salinity often results in poor stand establishment, reduced plant growth, and reduced yield of many horticultural crops such as peppers (*Capsicum annuum* L.) [29,30,31]. The best growth and number of leaves were observed in the control variant (0) and the AI variant, where salinity levels were recorded at 1.39 and 5.25 g NaCl/l, respectively.

**Figure 4:**
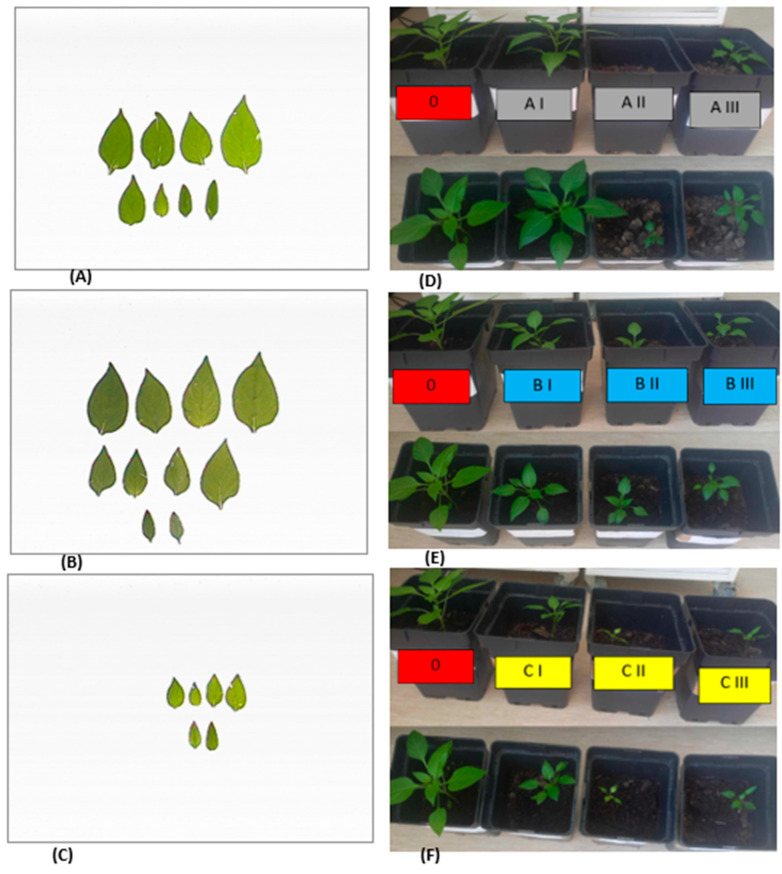
Leaves of seedling pepper variant: 0 (**A**), AI (**B**), and CII (**C**) on the 42nd day after seed sowing. Growth habit of seedling pepper plants produced on variant AI, AII, and AIII (**D**), BI, BII, and BIII (**E**), and CI, CII, and CIII (**F**).

Using composts A, B, and C combined with various fertilization doses results in a reduction in fresh mass. The type of compost shows no significant effect on fresh or dry mass, indicating comparable effectiveness among the three composts. The most favorable biometric parameters were exhibited by the pepper seedlings produced using substrate A (I). The fresh mass of these seedlings was 7.3% higher compared to the control variant. (Figure 3D). Additionally, the leaf area of pepper seedlings cultivated on substrate A (I) was 14% higher than that of the control variant (Figure 3E and Figure 4A). Despite containing only 25% compost A additive, this substrate resulted in the best plant growth. This suggests that the growth and mass of pepper seedlings were not directly regulated by the content of biogens in the tested substrates.

The conversion of organic nitrogen into nitrate involves enzymatic hydrolysis to ammonium, followed by bacterial oxidation to nitrate. This process relies on two key groups of aerobic soil bacteria: ammonia-oxidizing bacteria and nitrite-oxidizing bacteria [32]. Ultimately, nitrate concentration is the primary factor influencing growth rates. The slow conversion of ammonium to nitrate by bacteria in the substrate restricted the leaf area expansion of pepper seedlings (Figure 3F–H).

### 2.3. Nematode Analysis

Molecular metabarcoding has been proposed as a promising method to rapidly measure the community composition based on the genetic identification of species [33,34] (Table 1). Among plant parasitic nematodes, the following were found: *Ditylenchus persicus*, *Aphelenchidae*, *Aphelenchoides* sp. *K1 WY-2008*, *Belondoridae*, *Meloidogyne* spp., *Meloidogyne hispanica*, *Meloidogyne ethiopica*, and *Meloidogyne Lichtenburg*. The bacteriavorous were represented bythe following: *Dicranophoridae*, *Acrobeloides nanus*, *Panagrolaimus* cf. *rigidus AF40*, *Diplogasteroididae*, *Diplogasteridae*, *Pseudacrobeles curvatus*, *Neodiplogasteridae*, *Plectus acuminatus*, *Plectus* sp., *Plectus aquatilis*, *Chiloplectus andrassyi*, and *Plectidae*.

Among the fungivorous were *Aphelenchoides* spp. and *Aphelenchoides* sp. *K1 WY-2008*. Omnivores were represented by *Aporcelaimus* spp. and *Aporcelaimellus obtusicaudatus* and predatory species by *Daptonema*, *Aporcelaimus*, *Ichtyocephalidae*, and *Psyllotylenchus*.

Entomopathogenic nematodes were represented by *Oscheius* and *Oscheius onirici*.

**Figure 5:**
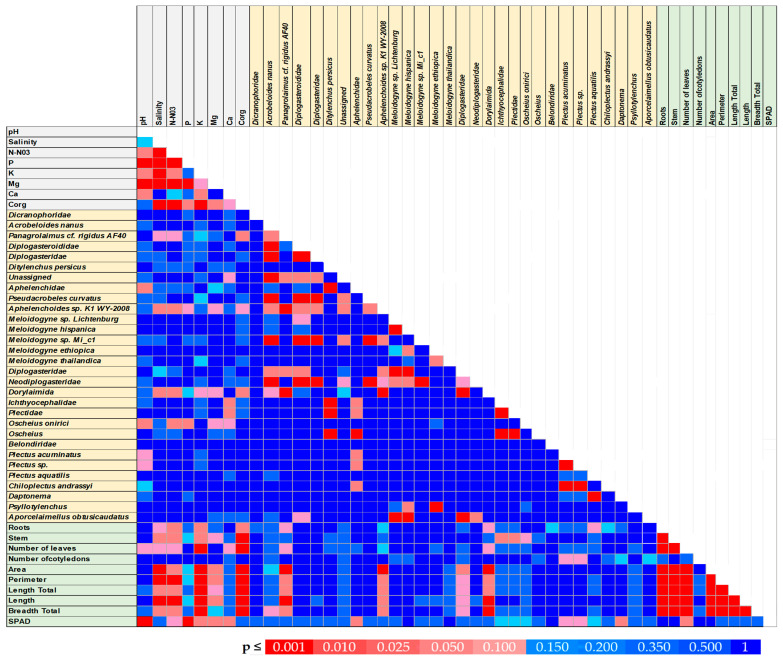
Colored p map for correlation coefficients between the parameters of the chemical properties of the substrates, physiological parameters of plants, and nematodes abundance. The correlation coefficients are significant with *p* < 0.05, n = 30.

The relationships between the individual components and the analyzed traits are illustrated in Figure 5.

The results of our study highlight the relationships between morphological traits (area, perimeter, length total, length, and breadth total) and SPAD (chlorophyll content) with the presence of nematode species: *Panagrolaimus* cf. *rigidus AF40* and *Aphelenchoides* sp. *K1 WY-2008* (Figure 5).

**Figure 6:**
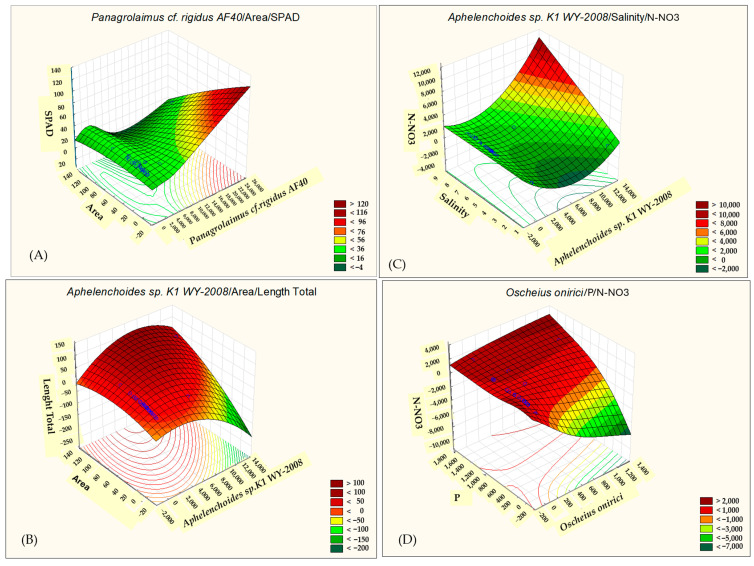
Correlation coefficients between the selected parameters of the analyzed traits: *Panagrolaimus* cf. *rigidus AF40*/area/SPAD (**A**), *Aphelenchoides* sp. *K1 WY-2008*/area/length total (**B**), *Aphelenchoides* sp. *K1 WY-2008*/salinity/N-NO_3_ (**C**), and *Oscheius onirici*/P/N-NO_3_ (**D**). The correlation coefficients are significant with *p* < 0.05.

The area exhibited the strongest positive correlations across these species, with coefficients of r = 0.701 for *Panagrolaimus* cf. *rigidus AF40* (Figure 6A) and r = 0.627 for *Aphelenchoides* sp. *K1 WY-2008* (Figure 6B). This trend may indicate that the nematode activity or abundance influences plant morphology, resulting in increased area of affected tissues or structures.

Other morphological parameters, such as length total, length, and breadth total, also showed moderate positive correlations. For instance, length correlated positively with *Panagrolaimus* cf. *rigidus AF40* (r = 0.578). Similarly, breadth total also demonstrated significant positive relationships.

In contrast, SPAD values, which reflect chlorophyll content, exhibited weak negative correlations with these nematode species. The correlation values were r = −0.129 for *Panagrolaimus* cf. *rigidus AF40*, (Figure 5 and Figure 6A) and r = −0.253 for *Aphelenchoides* sp. *K1 WY-2008* (Figure 5). Although the negative relationships were not strong, this trend indicates a potential reduction in chlorophyll content in plants associated with nematode presence. Such decreases could be due to physiological stress induced by nematode infestation, which may impair photosynthetic processes or cause tissue damage, leading to chlorophyll degradation. The weak negative correlations with SPAD imply that nematode presence may slightly reduce plant health or photosynthetic efficiency.

*Aphelenchoides* sp. *K1 WY-2008* exhibited negative correlations with salinity (r = −0.376) and nitrate nitrogen (N-NO_3_) (r = −0.415) (Figure 6B). These findings suggest that higher salinity and nitrate levels are associated with reduced presence or activity of this nematode species.

Similarly, *Oscheius onirici* was negatively correlated with pH (r = −0.439), but unlike the other species, it exhibited positive correlations with nitrate nitrogen (N-NO_3_) (r = 0.390) and phosphorus (P), (r = 0.362). These results indicate that *Oscheius onirici* thrives in nutrient-rich conditions, particularly where nitrate and phosphorus levels are elevated, despite lower pH levels (Figure 5 and Figure 6D).

*Panagrolaimus rigidus* represent bacteria feeders. The species of the genus *Aphelenchoides* spp. typically are fungivores, but several of them are plant *a* feeders, especially as migratory endoparasites inside roots and leaves (foliar nematodes) [17]. Those of economic importance in Polish agriculture include *A. ritzemabosi*, a pest affecting several vegetables and ornamental plants, as well as *A. fragariae*, which is problematic in strawberry fields.

*Oscheius onirici* belongs to the family Rhabditidae. These bacteria-feeding nematodes live in close association with insects [36,37,38]. *Oscheius* was divided into two groups: Insectivora and Dolichura. *O. onirici* spp. belongs to the Dolichura group [39].

Plant-parasitic nematodes (PPN), such as root-knot nematodes *(Meloidogyne* spp.) and cyst nematodes *(Heterodera* spp. and *Globodera* spp.), are important biotic stressors for many crops worldwide and are expected to be among the greatest threats to global food security in the future.

Root-knot nematodes (*Meloidogyne* spp.) are among the most destructive plant-parasitic nematodes, causing significant economic losses [40,41,42]. They are recognized as a major threat to pepper (*Capsicum annuum* L.) cultivation across the globe [43,44,45,46,47]. In pepper roots, the most common species are root-knot nematodes, including *Meloidogyne hapla*, *M. incognita*, *M. javanica*, and *M. arenaria* nematodes, as well as the potato cyst nematode *Globodera rostochiensis*. Infected plant roots exhibit nodular growths caused by root-knot nematodes or round, white or yellowish cysts formed as a result of nematode feeding. Nematode feeding impedes plant growth and can lead to a significant reduction in yield [48]. To prevent and control nematode infestations, it is recommended to avoid growing peppers and other solanaceous plants (such as potatoes, tomatoes, and eggplants) for 5–7 years. Crop rotation should be practiced, focusing on cereal crops followed by crops like onion and cucumber. Chemical disinfection of the substrate or the use of nematicides can effectively eliminate nematodes, a process that will be examined in the subsequent planned experiments.

The survival, reproduction, and population growth of *Meloidogyne* spp, like other plant-parasitic nematodes (PPNs), depend on various biotic and abiotic factors. These include consistent favorable temperature and moisture conditions, the continuous presence of suitable hosts with limited genetic variation, and minimal exposure to environmental extremes [49].

**Figure 7:**
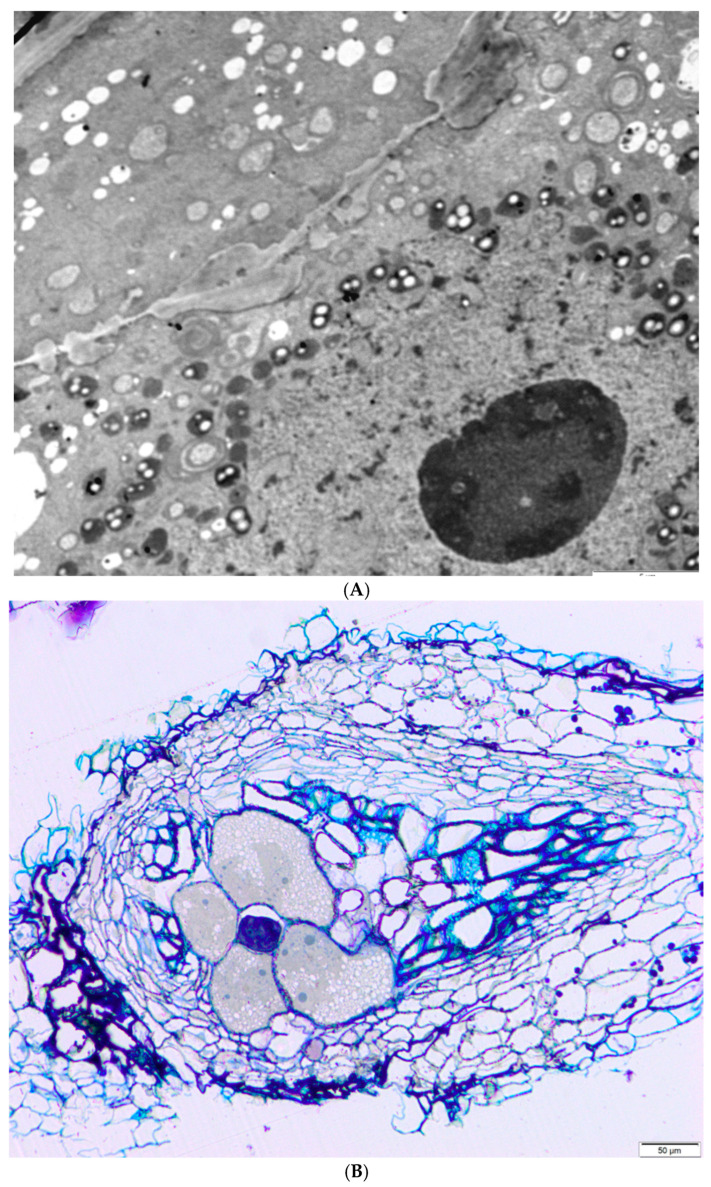
Electron microscope photographs of *M. hapla* giant cells in pepper root: (**A**,**B**). Bar = 5 μm (**A**), 50 μm (**B**). Author: Mirosław Sobczak, Warsaw University of Life Sciences.

Plant-parasitic nematodes play a key role among biotic factors, causing considerable economic losses and affecting their production. *Meloidogyne incognita* are the most common plant parasitic nematodes infesting *Capsicum* spp. crops [42,50,51]. Moens et al. [52] identify four primary species of *Meloidogyne* as significant: *M. arenaria*, *M. incognita*, and *M. javanica*—which are prevalent in tropical regions—and *M. hapla*, commonly found in temperate areas. Additionally, they highlight five emerging species in this genus: *M. chitwoodi*, *M. fallax*, *M. enterolobii*, *M. minor*, and *M. paranaensis*. A root-knot nematode species, *Meloidogyne thailandica* spp., was identified in 2002 on the roots of ginger (*Zingiber* spp.) from Thailand [53]. *M. incognita* can be regarded as having the greatest damage potential, since their presence in high numbers often results in significant yield losses. The feeding individuals remain with their whole bodies stationary in one area of the root/other belowground plant part and feed on giant cells. The galls disrupt the vascular system, leading to nutrient imbalances and, in some instances, compromising resistance in genotypes that are resistant to fungi and oomycetes [54]. Figure 7A shows pieces of two giant cells separated by an unevenly thickened cell wall. In the cytoplasm, there are numerous small vacuoles (white circles), plastids with starch grains (white dots in dark circles), and mitochondria (gray without white spots or in the shape of rings). The lower left corner is a folded cell nucleus with a huge nucleolus (large gray circle). Plastids and mitochondria are clustered around the nucleus.

**Figure 8:**
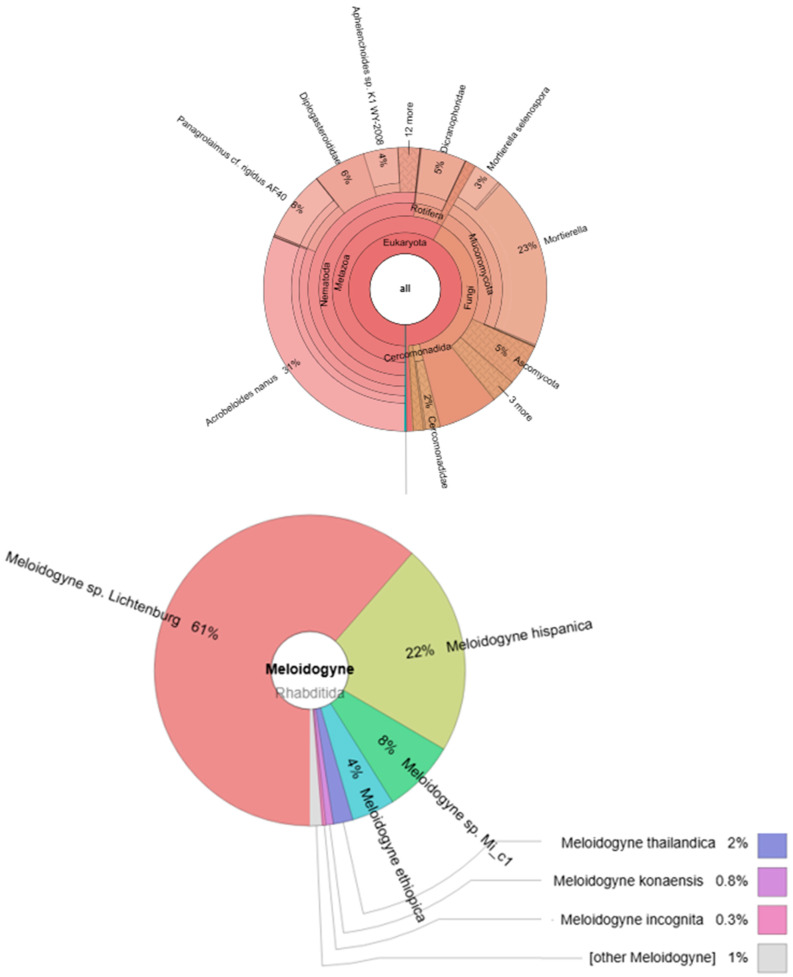
Krona graph representing the nematode community abundance estimated by NGS sequencing of pulled (n = 3) specimens in variant (0).

In our experiment, the presence of Meloidogyne was confirmed in different variants (AII, BI, BII, CII, and CIII). A total of 2250 individuals were also found in the control group: *Meloidogyne* sp. *Lichtenburg* (1566 individuals), *Meloidogyne hispanica* (562 individuals), *Meloidogyne* sp. *Mi_c1* (194 individuals), *Meloidogyne ethiopica* (114 individuals), *Meloidogyne thailandica* (52 individuals), *Meloidogyne konaensis* (20 individuals), *Meloidogyne incognita* (8 individuals), and other Meloidogyne (Figure 8).

Several plant-parasitic nematode (PPN) species are associated with diseases in pepper plants (*Capsicum annuum* L.). Among these, root-knot nematodes belonging to the genus Meloidogyne are the most commonly surveyed. The likelihood of *Meloidogyne javanica* [55,56] and *Meloidogyne arenaria* occurring in Poland is very low due to the low temperatures during the Polish growing season. *Meloidogyne incognita* is found in Polish climates only in greenhouses, while *Meloidogyne hapla* and *Meloidogyne hispanica* occur in open fields, as they are also present in Portugal and Spain [57,58]. Additionally, other PPN species, such as *Rotylenchus* spp. and *Ditylenchus* spp., may also exist in the rhizosphere of pepper plants.

The studies conducted by Robertson et al. [59], which examined 137 populations of *Meloidogyne incognita*, *Meloidogyne arenaria*, and *Meloidogyne hapla* from Spain and Uruguay parasitizing pepper, demonstrate that yield depends primarily on plant resistance and the race of the *Meloidogyne* species. *Meloidogyne hapla* was able to reproduce on resistant pepper plants, whereas no populations of *Meloidogyne javanica* or *Meloidogyne arenaria* reproduced on the same resistant plants. These findings have significant implications for designing alternative nematode control strategies using resistant pepper cultivars [60].

In southern European soils, peppers can also be affected by *Nacobbus aberrans* (false root-knot nematode), which induces similar galls on the roots [61]. The reproduction of this nematode has been reduced using soil disinfectants and nematophagous fungi. Various fungal species have been employed to control *Meloidogyne* spp. populations, including isolates of *Paecilomyces*, *Purpureocillium*, *Trichoderma*, *Fusarium solani*, *Talaromyces*, *Arthrobotrys dactyloides*, *Drechslerella*, and *Monacrosporium* [62]. Additional fungi used include *Pochonia chlamydospora* [63] and *Verticillium leptobactrum* [64], which has also been effective against *Globodera rostochiensis*. PPN nematodes can be also suppressed by nematophagous bacteria, and predatory nematodes like *Mononchoides composticola* [65,66] existed in compost products used as fertilizers in glasshouse pepper cultivars.

Pepper plants, like all crops, require a balanced supply of nutrients to thrive. Macronutrients such as nitrogen (N), phosphorus (P), and potassium (K) are essential for their vegetative growth and overall health. While phosphorus plays a crucial role in root development, potassium is vital for plant health, as it contributes to disease resistance and stress tolerance. The microenvironment in which plants grow has a significant impact on nematode populations. It would seem that higher doses of potassium could help protect plants from infections, particularly those caused by nematodes. Potassium is known to improve a plant’s resistance to various environmental stresses, including disease and drought. Research has shown that the application of potassium can reduce the occurrence of nematode infestations and, in turn, increase crop yields. For example, a study by Liu et al. [67] demonstrated that the treatment of rice with potassium sulfate (K_2_SO_4_) at low concentrations enhanced the plant’s defense mechanisms against *Meloidogyne* species. This suggests that potassium not only improves the plant’s overall health but also boosts its ability to fend off specific pests like root-knot nematodes. This finding has potential implications for the cultivation of peppers, where nematode control is crucial for maintaining high yields. In addition to potassium, the physical and chemical properties of the soil play an essential role in the development of nematode populations. Previous research by Al-Ghamdi [68] found that the concentration of several nutrients in the soil, including phosphorus, potassium, calcium, and magnesium, was positively correlated with the rate of nematode colonization on banana roots. As these nutrient levels increased, so did the number of nematodes, suggesting that nematodes thrive in soils with higher concentrations of these elements. On the other hand, an increase in nitrogen concentration appeared to reduce nematode populations, which may have implications for crop management strategies.

Soil health is closely linked to the presence and behavior of nematodes, as these organisms help indicate the status of soil fertility and its ability to support plant life. According to Gebremikael et al. [69], nematodes are not only indicators of soil health but also contribute to various soil functions, including nutrient cycling and organic matter decomposition. Therefore, understanding the interactions between soil nutrients and nematode populations is crucial for designing sustainable agricultural practices that promote both plant health and soil quality.

*Meloidogyne* can be introduced into an area through contaminated soil, plant tissues, or plant debris. The nematode has the potential to infest crops cultivated in soil-based substrates within plastic tunnels and greenhouses. Systematic cleaning and removal of plant residues from vehicles, machinery, and tools used in seedling production, plant cultivation, and maintenance are critical in preventing the spread of plant-parasitic nematodes and other harmful organisms.

In seedling production, substrates should be free from pathogenic organisms. When utilizing self-produced substrates, it is essential to disinfect them using either thermal or chemical methods to mitigate the risk of nematode infestation.

Nematodes represent the most abundant group of invertebrates in soil ecosystems, with densities ranging from 0.2 to 1 billion individuals per hectare. Approximately 25% of these nematodes are species that feed on plants and fungi. Brzeski [70] provides a comprehensive overview of plant-parasitic nematodes identified in Poland, highlighting challenges in distinguishing certain species. To date, around 260 species of herbivorous and mycophagous nematodes have been documented in Poland.

The economic significance of plant-parasitic nematodes lies in their capacity to impair plant growth and vitality, often leading to reduced yields or, in severe cases, total crop failure. Additionally, plants infected by nematodes exhibit increased susceptibility to secondary infections by other pathogens, including viruses, bacteria, and fungi. Among plant-feeding nematodes, several species are classified as quarantine pests, posing significant challenges to both domestic and international trade. The detection of quarantine-restricted nematode species can disrupt trade, necessitating stringent phytosanitary measures.

Given that Poland’s eastern border coincides with the European Union’s frontier, the Polish phytosanitary services play a critical role in safeguarding the EU against the introduction of harmful organisms through trade with non-EU countries. This responsibility underscores the importance of nematode management in protecting agricultural productivity and biosecurity across the region.

Understanding the relationship between soil nutrients and nematode populations is critical for managing nematode-related diseases. This relationship between soil nutrient composition and nematode activity highlights the importance of maintaining a balanced nutrient environment for plant health. Excessive or insufficient concentrations of certain nutrients can either encourage or hinder nematode infestations, underscoring the need for precise nutrient management in agriculture.

The bacterium *Pasteuria penetrans* is one of the most promising agents in the fight against *Meloidogyne arenaria*. Studies provided by De Leij at al. [71,72] shows the high effectiveness of this bacterium in controlling the J2 larvae of *Meloidogyne arenaria*. *Pasteuria penetrans*, by acting on the larvae, causes their destruction, thereby reducing the nematode population in the soil. The effectiveness of this bacterium makes it one of the potential tools in the biological control of root-knot nematodes.

Research by Kaplan and Noe [73] suggests that changes in soil composition, such as the addition of organic fertilizers, can also contribute to reducing nematode populations in the field. Specifically, the use of poultry manure for soil fertilization in tomato crops resulted in a decrease in the population of *Meloidogyne arenaria* and a reduction in the number of galls on the roots. These findings indicate that organic fertilizers may stimulate the growth of soil microorganisms that compete with nematodes for resources, leading to their natural suppression.

The bacterium *Bacillus penetrans* is another organism that shows potential in the biological control of nematodes. Research provided by Stirling [74] demonstrates that this bacterium, when applied at appropriate doses, effectively eliminates *Meloidogyne javanica*. The studies showed that when a dose exceeding five spores per J2 larva of *M. javanica* was used, more than 80% of the nematodes were infected, leading to their death. This confirms that *Bacillus penetrans* can be an effective tool for controlling certain nematode species, such as *Meloidogyne javanica.*

Current management practices are insufficient to fully control root-knot nematodes, and the use of certain chemicals has been increasingly restricted in recent years. As a result, it is essential to develop alternative control strategies that utilize environmentally friendly methods like application of biological control agents [75]. Biocontrol of root-knot nematodes has been used for decades, but it could still receive much more attention and achieve better results as new species are identified, characterized and evaluated for their efficacy against root-knot nematodes. Currently, targeted sequencing such as 16S and 18S rDNA sequencing has great potential to be applied for detection of new biological agents in root-knot nematodes management. This will make the biocontrol studies faster, cheaper, and more practical. Furthermore, it would be helpful to focus on the microbiomes of root-knot nematode-suppressive soils based on the metadata in future studies in order to explore possibilities for developing more holistic management strategies with multi-target modes of action.

## 3. Materials and Methods

### 3.1. Seed Material

In the experiment, certified seeds of the pepper variety ‘Marta Polka’ (PlantiCo, Zielonki, Poland), characterized by high germination capacity, were used. Marta Polka is known for their exceptional tolerance to adverse environmental conditions. This variety is known for its sweet yellow fruit intended for cultivation in the soil or in foil tunnels. This early plant grows up to 40 cm in height and produces rigid raised shoots, which are resistant to unfavorable weather conditions. This variety does not need to be staked in order to thrive.

### 3.2. Substrate Preparation Procedure

The composting process followed the methodology outlined in the study by Zapałowska et al. [76]. Compost was prepared using different organic matter in various mass proportions (% by mass):

A. Sewage sludge (0.80) + sawdust (0.20);
B. Sewage sludge (0.40) + sawdust (0.10) + biodegradable garden and park waste (0.50);
C. Biodegradable garden and park waste (0.90) + sawdust (0.10);

The organic mixture was subjected to the controlled composting process in plastic containers for 3 months. The moisture of the organic matter was monitored weekly. Temperature measurements confirmed the existence of four characteristic phases of the composting process, including mesophilic, thermophilic, cooling, and maturation phases.

Substrates for pepper seedling production were prepared as follows:

1. 0-control (100 O)
2. A I (0.75 A + 0.25 O);
3. A II (0.50 A + 0.50 O);
4. A III (0.25 A + 0.75 O);
5. B I (0.75 B + 0.25 O);
6. B II (0.50 B + 0.50 O);
7. B III (0.25 B + 0.75 O);
8. C I (0.75 C + 0.25 O);
9. C II (0.50 C + 0.50 O);
10. C III (0.25 C + 0.75 O).

### 3.3. Experimental Design

A controlled pot experiment was conducted using the prepared substrates for pepper seedling production. For this purpose, the prepared substrates were placed in production pots with a capacity of 0.5 L. Then, 3 seeds of the pepper variety ‘Marta Polka’ were sown at a depth of 1 cm in each pot. The pots were placed in a greenhouse with an air temperature of ±20 °C and a relative humidity of 80%. The moisture level in the substrate used for *Capsicum annuum* seedling production was maintained at 60% of the maximum water-holding capacity (MWHC). The experiment was performed in 3 replicates testing three types of compost variants (A, B, C) at four application levels (0%, 25%, 50%, 75%).

After 42 days from the sowing date, a series of measurements and analyses were performed to determine the impact of the applied substrates on the produced pepper seedling.

### 3.4. Analysis of Physicochemical Properties of Substrates

Analysis of the substrates was carried out according to the Polish Standard: PN-EN 12176:2004, PN-EN 13342:2002, PN-ISO 5656:2002, PBE-58 from 20.11.2015, PBE-58 from 20.11.2015, PN-R-04023:1996, PBE-24 ed. VI from 28.06.2007 [77]. The pH was determined using the potentiometric method. Salinity was assessed through electrical conductivity, while phosphorus and potassium levels were measured using the Egner–Riehm method. Magnesium was determined by the Schachtschabel method, and calcium and chlorine concentrations were analyzed using atomic absorption spectrophotometry. Total nitrogen was quantified by the Kjeldahl method, and total organic carbon was measured spectrophotometrically following oxidation. Nitrate (N-NO_3_) was determined using a colorimetric method with phenol-2,4 disulfonic acid [77].

### 3.5. Physiological Parameters of Plants

#### 3.5.1. The Relative Chlorophyll Content

The relative chlorophyll content in the leaves of pepper seedlings was measured using a SPAD 502 chlorophyll meter (SPAD; Minolta, Tokyo, Japan). The measurements were conducted on the 21st day after sowing and were replicated 20 times for each substrate variant.

#### 3.5.2. Biometric Parameters of Cotyledons and Leaves

The effect of the applied substrates for pepper seedling production on the size of developed plants was determined through measurements of selected biometric characteristics of seedlings The area and mass of cotyledons and leaves of produced pepper seedlings were assessed. Measurements of cotyledon and leaf area were conducted using a Leaf Area Meter- AM350 (ADC BioScientific Ltd., Global House, Geddings Road, Hoddesdon, Herts, UK) with 20 replications for each substrate variant. The results are presented in cm^2^. The mass of cotyledons and leaves was determined using a laboratory-scale RadWag WTB200 (RadWag, Radom, Poland). Individual cotyledons and leaves of pepper seedlings were separated from the rest of the plant and weighed in 20 replications for each substrate variant. The results are presented in grams.

### 3.6. Nematodes Extraction

A total of 200 cm^3^ of substrate variants was placed in a beaker, and the remaining water was added to achieve a total volume of 500 mL. The substrate was thoroughly mixed and allowed to settle at the bottom of the beaker. The sediment suspension was subsequently decanted and transferred to a 200 mL tube for centrifugation at 2000× *g* (RCF) for 3 min. The supernatant was then discarded, and the precipitate was resuspended with 80 mL of 1 molar sucrose solution [78].

The tubes were centrifuged again for 2 min at 2000× *g* (RCF). The supernatant, containing nematodes, was poured through a 25 μm sieve. Nematodes were extracted from the sieve and placed in glass containers with 30 mL capacity. Then, alive nematodes were analyzed by PCR procedure.

### 3.7. DNA Extraction and Libraries Preparation

Total DNA was extracted using a DNA Mini Kit (Syngen Biotech, Wrocław, Poland), following the manufacturer’s protocol. The D2-D3 segment of the 28S rDNA region was amplified using the specific primers. The PCR product was purified and a library was constructed using the NEBNext Multiplex Oligos for Illumina 96 Index Primers (New England Biolabs, Salisbury, UK). The resulting PCR products were pooled, and the final purified product was then quantified using qPCR according to the qPCR Quantification Protocol Guide (KAPA LibraryQuantification Kits for Illumina Sequencing platforms, Roche, Basel, Switzerland). The paired-end (300 bp with V3 chemistry) sequencing was performed using the MiSeq platform (Illumina, San Diego, CA, USA).

### 3.8. Processing and Analysis of Sequencing Data

The taxa of nematodes present in the analyzed samples were determined using the metabarcoding approach. Metabarcoding allows for simultaneous identification of many taxa within the same sample at all nematode developmental stages. The quality of the obtained reads was checked at FastQC and filtered using Trimmomatic to exclude low-quality reads (Q < 20, sequences with any ambiguous (N) bases, more than six homopolymers) and chimera sequences. Community composition and taxonomic affiliations of the obtained sequences were performed by the software pipeline CCMetagen v1.2.3. The processing and analysis of sequencing data were conducted using the criteria for taxonomic assignment in CCMetagen as follows: species-level similarity threshold of 99.00%, genus-level of 95.00%, family-level of 90.00%, order-level of 85.00%, class-level of 75.00%, and phylum-level of 55.00% [79].

### 3.9. Statistical Analysis

The results were statistically processed using the variance analysis in a 2-factor system of random blocks. The significance of differences with LSD of 0.05 was evaluated using Tukey’s multiple test. Statistical analysis of results was carried out using the ANALWAR-5.3 FR program by Franciszek Rudnicki (University of Life Science and Technology in Bydgoszcz, Poland). Pearson’s r correlation analysis was used to determine the relationship between continuous variables and to determine the relationship between quantitative variables (Statistica Version 13.3.0 by TIBCO, Palo Alto, CA, USA).

## 4. Conclusions

The findings underline the critical need for careful management of compost–peat mixtures, not only to enhance seedling growth but also to control the spread of harmful nematodes. To improve crop performance and minimize pest-related damage, it is crucial to prioritize the enhancement of substrate selection strategies. Further research is needed to develop effective methods for reducing nematode threats, such as *Meloidogyne* sp. and *Aphelenchoides* spp., in these substrates. Future studies should focus on developing and validating more efficient nematode control techniques, exploring alternative organic materials, and assessing the long-term impacts on plant health. These efforts could have practical applications in guiding agricultural practices toward the development of sustainable and pest-resistant substrate management strategies.

## Figures and Tables

**Table 1:** Nematode abundance in analyzed substrates. Species name according to Nemys: World Database of Nematodes ver. (11/2023); [35] GenBank (accessed on 28 November 2024).

Variant	1st Replicate	2nd Replicate	3rd Replicate
0	*Dicranophoridae* spp. 76%, *Acrobeloides nanus* 8%, *Panagrolaimus* cf. *rigidus AF40* 2%, *Diplogasteroididae* spp. 1%, *Ditylenchus persicus* 1%	*Diplogasteroididas* spp. 12%, *Aphelenchidae* spp. 7%, *Panagrolaimus* cf. *rigidus AF40* 4%, *Ditylenchus persicus* 4%, *Pseudacrobeles curvatus* 6%, *Acrobeloides nanus* 54%	*Panagrolaimus* cf. *rigidus AF40* 15%, *Diplogasteroididae* spp. 12%, *Aphelenchoides* spp. *K1 WY-2008* 8%, *Meloidogyne* spp. *Lichtenburg* 1%, *Diplogasteridae* spp. 1%, *Acrobeloides nanus* 60%
	(1)	(2)	(3)
AI	*Ditylenchus persicus* 64%, *Acrobeloides nanus* 15%, *Oscheius* spp. 13% *Aphelenchidae* spp. 4% *Ichthyocephalidae* spp. 0.8%, *Plectidae* spp. 2%	*Acrobeloides nanus* 50%, *Dicranophoridae* spp. 50%	*Pseudacrobeles curvatus* 9%, *Ditylenchus persicus* 14%, *Pseudacrobeles curvatus* 9%, *Acrobeloides nanus* 76%
	(4)	(5)	(6)
AII	*Oscheius onirici* 23%, *Acrobeloides nanus* 19%, *Belondiridae* spp. 19%, *Ditylenchus persicus* 40%	*Dicranophoridae* spp. 2%, *Acrobeloides nanus* 24%, *Panagrolaimus* cf. *rigidus AF40* 23%, *Ditylenchus persicus* 15%, *Oscheius onirici* 3%, *Meloidogyne* sp. *Lichtenburg* 1%, *Aphelenchidae* spp. 26%	*Ditylenchus persicus* 24%, *Acrobeloides nanus* 19%, *Diplogasteroididae* 12%, *Oscheius onirici* 8%, *Aphelenchidae* spp. 5%, *Meloidogyne* sp. *Lichtenburg* 2%, *Panagrolaimus* cf. *rigidus AF40* 26%
	(7)	(8)	(9)
AIII	*Panagrolaimus* cf. *rigidus AF40* 6%, *Aphelenchoididae* spp. 33%, *Aphelenchoides* spp. 0.9%, *Plectus acuminatus* 0.9%, *Plectus* spp. 2%, *Chiloplectus andrassyi* 23%	*Daptonema* spp. 5%, *Acrobeloides nanus* 5%, *Panagrolaimus* cf. *rigidus AF40* 15%, *Plectus acuminatus* 1%, *Plectus* spp. 2%, *Plectus aquatilis* 10%	*Diplogasteroididae* spp. 13%, *Neodiplogasteridae* spp. 1%, *Diplogasteridae* spp. 1%, *Acrobeloides nanus* 83%
	(10)	(11)	(12)
BI	*Meloidogyne* sp. *Lichtenburg* 21%, *Meloidogyne hispanica* 4%, *Meloidogyne* sp. *Mi_c1* 3%, *Meloidogyne ethiopica* 2%, *Acrobeloides nanus* 15%, *Pseudacrobeles curvatus* 5%, *Panagrolaimus* cf. *rigidus AF40* 7%, *Psyllotylenchus* spp. 2%, *Aphelenchidae* spp. 2%, *Oscheius oniric* 2%, *Ditylenchus persicus* 34%	*Panagrolaimus* cf. *rigidus AF40* 19%, *Acrobeloides nanus* 33%, *Belondiridae* spp. 49%	*Ichthyocephalidae* spp. 7%, *Neodiplogasteridae* spp. 5%, *Pseudacrobeles curvatus* 11%, *Acrobeloides nanus* 60%, *Dicranophoridae* spp. 17%
	(13)	(14)	(15)
BII	*Acrobeloides nanus* 26%, *Oscheius onirici* 8%	*Acrobeloides nanus* 37%, *Dicranophoridae* spp. 57%, *Ditylenchus persicus* 5%	*Diplogasteroididae* spp. 28%, *Meloidogyne* sp. Lichtenburg 8%, *Meloidogyne hispanica* 1%, *Ditylenchus persicus* 5%, *Neodiplogasteridae* spp. 3%, *Diplogasteridae* spp. 2%, *Aporcelaimellus obtusicaudatus* 0.1%, *Acrobeloides nanus* 47%
	(16)	(17)	(18)
BIII	*Ditylenchus persicus* 3%, *Panagrolaimus* cf. *rigidus AF40* 3%, *Pseudacrobeles curvatus* 3%, *Acrobeloides nanus* 46%	*Acrobeloides nanus* 15%, *Oscheius* spp. 13%, *Aphelenchidae* spp. 4%, *Ditylenchus persicus* 64%	*Diplogasteroididae* spp. 27%, *Diplogasteridae spp.* 3%, *Panagrolaimus* cf. *rigidus AF40* 3%, *Aphelenchoides* sp. *K1 WY* 1%, *Acrobeloides nanus* 59%
	(19)	(20)	(21)
CI	*Acrobeloides nanus* 63%	*Diplogasteroididae* spp. 21%, *Aphelenchidae* spp. 14%, *Pseudacrobeles curvatus* 11%, *Acrobeloides nanus* 39%	*Acrobeloides nanus* 51%
	(22)	(23)	(24)
CII	*Aphelenchidae* spp. 18%, *Ditylenchus persicus* 2%, *Diplogasteroididae* spp. 1%, *Meloidogyne* sp. *Lichtenburg* 1%, *Panagrolaimus* cf. *rigidus AF40* 1%, *Pseudacrobeles curvat* 11%, *Acrobeloides nanus* 48%	*Panagrolaimus* cf. *rigidus AF40* 5%, *Diplogasteroididae* spp. 5%, *Acrobeloides nanus* 54%,	*Panagrolaimus* cf. *rigidus AF40* 1%, *Plectus aquatilis* 1%, *Chiloplectus andrassyi* 0.8%
	(25)	(26)	(27)
CIII	*Diplogasteroididae* spp. 26%, *Meloidogyne* sp. *Mh_c1* 2%, *Diplogasteridae* spp. 3%, *Aphelenchoides* sp. *K1 WY-2008* 2%, *Neodiplogasteridae* spp. 2%, *Pseudacrobeles curvatus* 11%, *Acrobeloides nanus* 37%	*Diplogasteroididae* spp. 35%, *Acrobeloides nanus* 43%, *Aphelenchidae* spp. 22%	*Oscheius* spp. 36%, *Acrobeloides nanus* 14%, *Panagrolaimus* cf. *rigidus AF40* 6%, *Meloidogyne* sp. *Lichtenburg* 26%, *Meloidogyne hispanica* 5%, *Meloidogyne* sp. *Mi_c1* 3%, *Meloidogyne ethiopica* 2%, *Meloidogyne thailandica* 1%
	(28)	(29)	(30) *

* The data can be found in the Supplementary Materials as follows: 0_1.HTML Document (1); 0_2.HTML Document (2); 0_3.HTML Document (3); AI_1.HTML Document (4); AI_2.HTML Document (5); AI_3.HTML Document (6); AII_1.HTML Document (7); AII_2.HTML Document (8); AII_3.HTML Document (9); AIII_1.HTML Document (10); AIII_2.HTML Document (11); AIII_3.HTML Document (12); BI_1.HTML Document (13); BI_2.HTML Document (14); BI_3.HTML Document (15); BII_1.HTML Document (16); BII_2.HTML Document (17); BII_3.HTML Document (18); BIII_1.HTML Document (19); BIII_2.HTML Document (20); BIII_3.HTML Document (21); CI_1.HTML Document (22); CI_2.HTML Document (23); CI_3.HTML Document (24); CII_1.HTML Document (25); CII_2.HTML Document (26); CII_3.HTML Document (27); CIII_1.HTML Document (28); CIII_2.HTML Document (29); CIII_3.HTML Document (30).

## Data Availability

Data are only available upon request due to restrictions.

## Supplementary Materials

The following supporting information can be downloaded at: https://www.mdpi.com/article/10.3390/ijms26020442/s1.
