# Supplementary material for: Optimization of Compost and Peat Mixture Ratios for Production of Pepper Seedlings
Source: Int J Mol Sci. 2025 Jan 7;26(2):442. doi: 10.3390/ijms26020442 (PMC11765180; doi:10.3390/ijms26020442)
Supplement: Supplementary file 1 [file ijms-26-00442-s001.zip › CC_metagen_1.3 server_results/0_1.html]

Javascript must be enabled to view this page.

magnitude
magnitudeUnassigned

results

10936

10868
20

116

80

80

80

36

36

36

1782

222

222

222

38

38

38

22

22

22

138

138

138

24

210

210

96

96

96

114

1350

1350

1350

1350

16

8864
1812

634

634

634

634

634
106

528

3794

894

894

772

772

772

122

122

202

202

202

202

202

1870

20

20

20

20

20

178

176

176

176

176

454

344

150

150

150

52

52

118

118

118

24

24

24

84

84

84

84

26

26

2548

672

32

32

32

32

570

460

20

20

22

418

110

110

36

36

36

34

34

34

390

390

390

390

24

366

1282

1282

1282

136

136

136

136

136

40

28

28

28

76

70

70

24

24

24

24

46

46

46

46

46

68

68
