# Supplementary material for: Optimization of Compost and Peat Mixture Ratios for Production of Pepper Seedlings
Source: Int J Mol Sci. 2025 Jan 7;26(2):442. doi: 10.3390/ijms26020442 (PMC11765180; doi:10.3390/ijms26020442)
Supplement: Supplementary file 1 [file ijms-26-00442-s001.zip › CC_metagen_1.3 server_results/0_2.html]

Javascript must be enabled to view this page.

magnitude
magnitudeUnassigned

results

880

880

692

390
52

338

338

28

16

16

16

14

14

14

234

210

210

24

24

46

302

302

302

302

188

88

88

88

88

88

8

8

8

8

8

8

92
