# Supplementary material for: Optimization of Compost and Peat Mixture Ratios for Production of Pepper Seedlings
Source: Int J Mol Sci. 2025 Jan 7;26(2):442. doi: 10.3390/ijms26020442 (PMC11765180; doi:10.3390/ijms26020442)
Supplement: Supplementary file 1 [file ijms-26-00442-s001.zip › CC_metagen_1.3 server_results/0_3.html]

Javascript must be enabled to view this page.

magnitude
magnitudeUnassigned

results

307630

307164
2342

66

66

5886

718

718

5168
430

4738

179188

159434
338

159006

159006
62

52

12098

12084

12084

14

14

1444

664
572

92

92

42

18420

102

140

24490

10

10

52

24428

10

24418

2550

2550
34

114

562

8

52

1566

20

194

216

216

58

58

680

680

1620

130

130

96238
36

320

320

95596
52

108

95436

64

130

40

52

90

90

58

58

58

32

32

32

3712

20

20

20

20

3440

3440

3440

252

48

48

204

204

204

204

16

16

16

16

16

16026
110

15916

15916

48

18

18

18

15850

24

116140
20966

26

26

26

26

72030
26

71802

71802

71802

362

146

146

71294
60662

9688

58

60

826

202

202

202

202

58

144

14578
268

4720

4720

3534

44

34

22

26

26

3408

3408

1186

1186

1254

1254

20

20

1210

1154

1154

56

24

24

24

46

46

46

46

794

794

794
202

492

100

100

2064

884

354

174

24

24

190

100

90

90

20

20

20

20

122

122

3096

2828

2720

212

212

18

18

2490
122

2368

46

24

22

22

20

20

42

42

108

108

108

76

76

20

20

56

56

40

40

44

44

270

108

62

62

62

46

46

46

162

120

120

120

42

166

936

510

510

510

26

26

246

246

154

60

60

60

58

58

58

36

36

80

80

80

80

80

4388
170

62

62

62

2316

80

50

50

50

30

30

30

1130

1130

530

56

474

282

282

318

130

130

36

36

56

56

38

212

764

764

764

1198

1198

1198

1198

278

920

172

58

282

88

32

32

32

32

162

162

162

162

106

106

106

24

24

24

4152

280

280

54

54

54

226

226

226

554
44

510

70

70

70

440
32

408

408

768

768

768

768

1916

1916
1858

34

24

466
