# Supplementary material for: Optimization of Compost and Peat Mixture Ratios for Production of Pepper Seedlings
Source: Int J Mol Sci. 2025 Jan 7;26(2):442. doi: 10.3390/ijms26020442 (PMC11765180; doi:10.3390/ijms26020442)
Supplement: Supplementary file 1 [file ijms-26-00442-s001.zip › CC_metagen_1.3 server_results/AIII_1.html]

Javascript must be enabled to view this page.

magnitude
magnitudeUnassigned

results

20432

20432
40

8706

8706

932

932

932

7774

7774

44

44

44

132

132

132

132

10116

9364
918

7646

3924

3924

3484
1348

2136

378

216

46

84

32

62

3722

604

604

604

3082
2988

86

8

8

36

36

36

348

40

40

308

134

134

174

174

174

452

40

40

40

408

408

272

272

272

272

272

32

32

32

32

20

20

20

20

20

1026
220

50

50

50

50

50

50

442

148

148

148

148

148

74

74

74

74

40

170

170

170

170

170

10

10

10

10

10

254

86

86

86

86

86

110

110

110

110

110

58

60

348

348

18

18

18

18

330

330

330

330
