# Supplementary material for: Optimization of Compost and Peat Mixture Ratios for Production of Pepper Seedlings
Source: Int J Mol Sci. 2025 Jan 7;26(2):442. doi: 10.3390/ijms26020442 (PMC11765180; doi:10.3390/ijms26020442)
Supplement: Supplementary file 1 [file ijms-26-00442-s001.zip › CC_metagen_1.3 server_results/AIII_2.html]

Javascript must be enabled to view this page.

magnitude
magnitudeUnassigned

results

2574

2574

2398

2298
158

1182

592

592

592
288

222

48

34

486

344

344

344

142

124

124

18

18

104

104

104

958

868

420

420

448

90

90

38

38

38

38

62

62

62

62

176

44

44

44

44

44

44

16

16

16

16

10

10

6

6

40

40

40

40

40

24

24

24

24

24

52
