# Supplementary material for: Optimization of Compost and Peat Mixture Ratios for Production of Pepper Seedlings
Source: Int J Mol Sci. 2025 Jan 7;26(2):442. doi: 10.3390/ijms26020442 (PMC11765180; doi:10.3390/ijms26020442)
Supplement: Supplementary file 1 [file ijms-26-00442-s001.zip › CC_metagen_1.3 server_results/AIII_3.html]

Javascript must be enabled to view this page.

magnitude
magnitudeUnassigned

results

155420

155354
254

66

66

1856

174

174

1682
344

1338

133244

27886
170

27496

56

56

56
10

46

27440

86

86

86

278

22

22

22

50

268

23076

23034

26

23008

12

30

3660

220

122

110

12

98

22

22

22

76

76

76

862

40

40

40

40

822

822

822

104458
410

104048

104048

142

16

16

16

103880

10

38

38

38

38

38

38

22

18708
6300

2474

2182

2182

2182

52

52

24

2106

2106

292

292

134

134

134

158

158

32

26

100

1998

100

100

74

74

26

26

26

720

720

598

572

572

26

20

20

20

102

102

102

68

38

38

38

38

30

30

30

30

156

74

74

74

82

82

82

82

50

482

62

330

330

330

330

90

90

422

394

330

330

330

64

64

28

28

5980

28

28

28

4930

68

68

30

30

38

236

236

54

54

30

30

152

114

4512

4512

4512

984

984

984

984

74

910

18

20

1956

102

102

54

54

54

18

18

18

30

30

30

30

1012
112

900

900
94

806

36

36

36

36

54

54

54

66

66
