# Supplementary material for: Optimization of Compost and Peat Mixture Ratios for Production of Pepper Seedlings
Source: Int J Mol Sci. 2025 Jan 7;26(2):442. doi: 10.3390/ijms26020442 (PMC11765180; doi:10.3390/ijms26020442)
Supplement: Supplementary file 1 [file ijms-26-00442-s001.zip › CC_metagen_1.3 server_results/AII_1.html]

Javascript must be enabled to view this page.

magnitude
magnitudeUnassigned

results

394

394

296

248

202

202

98

98

98

48

48

48

56

56

56

46

46

46

48

48

48

48

98

48

48

48

12

12

12

36

18

18

18

20

20

20

20

20

30
