# Supplementary material for: Optimization of Compost and Peat Mixture Ratios for Production of Pepper Seedlings
Source: Int J Mol Sci. 2025 Jan 7;26(2):442. doi: 10.3390/ijms26020442 (PMC11765180; doi:10.3390/ijms26020442)
Supplement: Supplementary file 1 [file ijms-26-00442-s001.zip › CC_metagen_1.3 server_results/AII_2.html]

Javascript must be enabled to view this page.

magnitude
magnitudeUnassigned

results

9890

9890

90

90

90

90

9066

8776

8776

18

18

18

8758

2396

26

1398
26

1372

1372

316

2050

2050

2050

134

134

22

112

248

248

248

2190

2190

2190

80

80

80

210

210

210

210

688
30

66

66

66

66

66

66

222

24

24

24

24

62

136

136

136

136

136

298

176

176

176

176

122

122

122

122

122

72

46

46

46

46

46
