# Supplementary material for: Optimization of Compost and Peat Mixture Ratios for Production of Pepper Seedlings
Source: Int J Mol Sci. 2025 Jan 7;26(2):442. doi: 10.3390/ijms26020442 (PMC11765180; doi:10.3390/ijms26020442)
Supplement: Supplementary file 1 [file ijms-26-00442-s001.zip › CC_metagen_1.3 server_results/AII_3.html]

Javascript must be enabled to view this page.

magnitude
magnitudeUnassigned

results

34854

34626
34

100

100

100
30

70

198

26

26

172

172

172

22052

15148

15066

16

16

16

16

15050

778

116

3724
38

3686

3686

1758

3920

3920

3920

526

526

20

84

16

368

38

118

1258

1258

1258

2852

2852

2852

82

26

26

26

26

56

56

48

48

48

48

6856
22

6834

6834

6834

52

52

52

52

12144
384

1324

1324

1324

1324

78

78

1246
22

1170

54

3154
22

1200

1200

348

348

852

852

852

216

216

216

216

216

20

20

20

20

20

36

56

56

56
28

28

220

26

26

26

26

26

1358

1006

1006

1006

1006

158

158

158

158

102

102

92

92

930

424

18

406

406

406

506

506

506

506

34

472

6352

46

46

46

228
