# Supplementary material for: Optimization of Compost and Peat Mixture Ratios for Production of Pepper Seedlings
Source: Int J Mol Sci. 2025 Jan 7;26(2):442. doi: 10.3390/ijms26020442 (PMC11765180; doi:10.3390/ijms26020442)
Supplement: Supplementary file 1 [file ijms-26-00442-s001.zip › CC_metagen_1.3 server_results/AI_1.html]

Javascript must be enabled to view this page.

magnitude
magnitudeUnassigned

results

101340

101306
344

418

418

146

146

146

272

272
28

244

2348

120

120

2228
194

2034
30

2004

82132

80944
190

80584

1232

1232

79352
22

3100

52102
22

52080

164

51916

72

72

72

32

630

240

124

32

92

116

116

230

230

46

184

94

10798
24

10774

12032

12016

16

12000

16

16

170

170

138

32

32

32

30

30

30

30

30

528

44

18

18

18

26

26

26

484

36

36

36

16

16

16

432

630
46

584

584

584

100

15822
328

1278
22

1256

1256

1256

1256

1256

7346

288

288

102

102

102

118

118

118

68

68

1956

1956

1918

1882

1882

36

38

38

38

28

28

28

28

482

482

482
438

44

308

544

80

44

38

24

24

14

382

382

382

3040

2306

2150

2150
58

2092

44

24

20

20

112

112

142

142

22

120

120

514

28

28

28

296

296

190

190

190

78

78

268

56

56

56

56

46

46

46

46

166

166

166

166

270

162

162

162

162

5106

482

46

46

46

46

204

204

178

178

26

114

114

114

22

22

22

22

96

4304

4304

4304

4304

180

4124

158

158

158

158

158

162

64

64

64

64

98

38

38

38

60

60

1764

88

24

24

24

24

64

64

64

64

54

54

54

34

34
