# Supplementary material for: Optimization of Compost and Peat Mixture Ratios for Production of Pepper Seedlings
Source: Int J Mol Sci. 2025 Jan 7;26(2):442. doi: 10.3390/ijms26020442 (PMC11765180; doi:10.3390/ijms26020442)
Supplement: Supplementary file 1 [file ijms-26-00442-s001.zip › CC_metagen_1.3 server_results/AI_2.html]

Javascript must be enabled to view this page.

magnitude
magnitudeUnassigned

results

186

186

108

54

54

54

54

54

54

54

54

54

54

78

38

38

38

38

38

38

40
