# Supplementary material for: Optimization of Compost and Peat Mixture Ratios for Production of Pepper Seedlings
Source: Int J Mol Sci. 2025 Jan 7;26(2):442. doi: 10.3390/ijms26020442 (PMC11765180; doi:10.3390/ijms26020442)
Supplement: Supplementary file 1 [file ijms-26-00442-s001.zip › CC_metagen_1.3 server_results/AI_3.html]

Javascript must be enabled to view this page.

magnitude
magnitudeUnassigned

results

244714

244226
140

82

82

82

82
40

42

72

72

72

72

72

424

182

242

242

189554

41078
176

40876

56

56

40820
22

18

18

18

5872

5872

22

5850

34908

31192

34

31158

3602

3602

40

44

30

26

26

26

146050

145758

28

28

28

28

145730
145562

168

102

66

66

292

292

292

292

2426
36

2390

2390

2390

53688
11860

50

50

50

50

50

3494

3494

3494

3494

54

54

88

3352
452

2768

98

34

9752
260

2070

2070

16

2054

884

884

1142

28

34

34

560

560

560
334

128

98

98

302

676

46

78

216

44

44

44

40

40

132

132

132

336

336

336

336

2718

2468

1476

1476
238

1238

66

66

890

28

862

862

36

36

20

20

20

20

230

74

74

74

62

62

62

94

94

524

490

24

24

24

466

366
318

18

30

100
20

34

46

34

34

1350

1210

270

270

270

164

164

36

36

36

740

740

740

740

48

28476
44

80

48

48

32

32

6130

458

212

212

212

176

176

30

40

40

124

124

124
28

96

4982

4882
30

80

798
582

216

138

3836

100

100

244

244

322

322

322

848

848

848

848

52

796

720
42

678

678
258

420

420

68

64

64

64

19792

19792

19792

138

254

32

32

180

180

180

180

42

42

42

338

338

50

288

56

206

206

26

26

26

26

26

158

158

118

118

40

40

22

22

22

22

60

60

60

488

488
