# Supplementary material for: Optimization of Compost and Peat Mixture Ratios for Production of Pepper Seedlings
Source: Int J Mol Sci. 2025 Jan 7;26(2):442. doi: 10.3390/ijms26020442 (PMC11765180; doi:10.3390/ijms26020442)
Supplement: Supplementary file 1 [file ijms-26-00442-s001.zip › CC_metagen_1.3 server_results/BIII_1.html]

Javascript must be enabled to view this page.

magnitude
magnitudeUnassigned

results

374382

374292
962

20

20

20

20

20

20

192

192

1456

356

1100
72

1028

329154

211752
362

210560

340

340

92
24

68

248

196

32

20

210220

832

7372

7372

7372

130

130

130

34

298

20

20

278

8

270

12

12

12

546

118

118

200820
60

30

108

178

184298
64

36

194

184004

15852

15852

30

206

58

58
32

26

26

830

688

486

180
162

18

22

108

24

24

24

64

64

64

20

20

34

34

34

34

4384

34

34

34

34

4350

4350

4228

4228

4228

122

86

86

36

36

26

26

26

22

22

22

22

22

22

112970
652

112318

112318

200

482
468

14

14

111618

18

48

26

39318
1066

148
106

42

42

42

27784

27784

27784

27784

112

27672
24556

3032

84

6004
88

48

48

20

20

20

28

28

28

868

868

40

40

40

708

708

708

86

22

22

64

64

34

34

34

180

180

180

92

88

88

408

1862

108

108

48

276

330

124

92

32

90

68

22

22

116

116

1100

1100

44

1056

1138

990

978

978
280

698

12

12

124

124

124

24

24

176

176

112

112

112

64

64
20

44

468

768

122

374

374

32

342

190

190

82
44

20

20

18

18

18

56

2766
112

62

62

62

138

34

34

104

104

508

16

16

126

126

126

266

266

230

230

36

36

66

66

66

66

34

34

34

912

912

912

912

254

658

94

94

94

94

94

18

18

18

18

18

138

62

62

76

76

76

76

474

474

442

32

32

310

22

22

22

22

20

20

18

18

18

18

26

26

64

32

32

32

32

32

32

160

32

32

32

72

72

72

20

20

20

8

28

28

28

166

1328

456

424

14

14

14

248

248

248

38

38

38

36

36

36

88

88

32

32

32

32

32

36

36

36

36

36

36

1416
142

1274

1274
362

912

844

68

44

44

40

40

1084

1084

1084

1084

40

40

40

90

90
