# Supplementary material for: Optimization of Compost and Peat Mixture Ratios for Production of Pepper Seedlings
Source: Int J Mol Sci. 2025 Jan 7;26(2):442. doi: 10.3390/ijms26020442 (PMC11765180; doi:10.3390/ijms26020442)
Supplement: Supplementary file 1 [file ijms-26-00442-s001.zip › CC_metagen_1.3 server_results/BIII_3.html]

Javascript must be enabled to view this page.

magnitude
magnitudeUnassigned

results

10500

10500

26

26

26

26

36

36

36

36

9688

2136

2112

14

14

14

14

2098

44

54

54

54

30

30

30

58

64

1270

1270

1270

578

24

24

24

24

7552
24

7528

7528

7528

750
190

256

256

256

256

256

256

160

104

104

104

82

82

22

56

56

30

30

30

26

26

22

22

22

22

22

22

122
