# Supplementary material for: Optimization of Compost and Peat Mixture Ratios for Production of Pepper Seedlings
Source: Int J Mol Sci. 2025 Jan 7;26(2):442. doi: 10.3390/ijms26020442 (PMC11765180; doi:10.3390/ijms26020442)
Supplement: Supplementary file 1 [file ijms-26-00442-s001.zip › CC_metagen_1.3 server_results/BII_1.html]

Javascript must be enabled to view this page.

magnitude
magnitudeUnassigned

results

612

612

280

96

96

96

74

74

74

22

22

22

20

20

20

20

118

118

118

118

118

118

46

46

46

46

332

60

60

60

60

60

60

42

42

42

16

16

16

26

16

16

10

164

164

164

164

164

66
