# Supplementary material for: Optimization of Compost and Peat Mixture Ratios for Production of Pepper Seedlings
Source: Int J Mol Sci. 2025 Jan 7;26(2):442. doi: 10.3390/ijms26020442 (PMC11765180; doi:10.3390/ijms26020442)
Supplement: Supplementary file 1 [file ijms-26-00442-s001.zip › CC_metagen_1.3 server_results/BII_2.html]

Javascript must be enabled to view this page.

magnitude
magnitudeUnassigned

results

77730

77682
378

54

54

694

162

162

532
90

442

442

56172

23818

23818

23818

2956

2956

2956

20862

20746

20746

106

106

10

188

188

188

188

32166
158

32008

32008

52

31956

16

18762
360

44

44

44

44

44

44

13474

13474

13474

13474
308

13166
11624

1542

2568
160

874

874

766

766

766

22

22

86

86

86

22

154

118

118

118

118

36

36

36

66

66

66

114

764

64

38

38

26

26

26

628

628

72

72

414

394

350

350
120

230

44

44

20

20

20

20

1820

76

76

76

904

234

234

82

82

36

36

116

58

58

34

34

24

612

612

612

574

574

574

574

108

18

448

40

40

40

20

26

26

180

180

180

496

322

322

34

34

34

142

142

142

18

18

18

128

128

128

128

128

510
86

424

424

424
138

286

774

774

774

774

48

48
