# Supplementary material for: Optimization of Compost and Peat Mixture Ratios for Production of Pepper Seedlings
Source: Int J Mol Sci. 2025 Jan 7;26(2):442. doi: 10.3390/ijms26020442 (PMC11765180; doi:10.3390/ijms26020442)
Supplement: Supplementary file 1 [file ijms-26-00442-s001.zip › CC_metagen_1.3 server_results/BII_3.html]

Javascript must be enabled to view this page.

magnitude
magnitudeUnassigned

results

248852

248226
1454

92

92

6072

610

610

5462
196

5266

115990

107352
514

106728

106728
132

80

3386

7090
1346

5744

20

5724

29730

182

70

58

12

12

11872
44

11828
46

38

1426

290

40

78

40

26

664

22

8336

14

808

274

274

48

48

2442

51302
36

600

600

50458

38

52

50368

38

20

80

36

34

120

120

120

110

110

110

110

110

410

18

18

18

18

392

36

36

36

36

356

356

8208
72

8092

8092

8092

44

44

44

20

20

20

20

20

20

24

24

24

24

24

28

123606
28214

28

28

28

28

58472

58440

58440

58440

516

126

126

57798
49774

7584

440

32

32

32

32

32

13492
334

1362

1362

1064

24

24

1040

1040

298

298

640

640

522

522

118

118

118

600

600

600
154

446

410

1954

190

192

352

294

294

294

30

30

28

28

1220

24

1196

1196

7374

10

10

10

10

5374

5026

60

60

42

42

20

20

44

44

4860
50

4810

134

62

20

52

52

92

92

46

46

76
28

48

48

138

138

138

138

228

228

24

50

50

154

154

52

52

1476

1476

96

96

160

36

36

36

36

36

592

316

316

316

52

52

142

142

82

82

82

82

30

30

30

30

30

4120
210

26

26

26

1916

78

78

78

78

1138

1138

1102

190

912

36

28

28

28

58

614

614

614

1512

1512

1512

1512

564

948

54

108

108

108

108

108

88

88

88

88

88

138

20

20

20

20

118

118

28

28

28

40

40

40

40

19280

142

142

44

44

44

56

34

34

34

34

22

22

22

22

42

42

16

16

26

432
60

372

88

88

88

284

284

284

160

160

160

160

84

84

84

84

142

142

114

28

626
