# Supplementary material for: Optimization of Compost and Peat Mixture Ratios for Production of Pepper Seedlings
Source: Int J Mol Sci. 2025 Jan 7;26(2):442. doi: 10.3390/ijms26020442 (PMC11765180; doi:10.3390/ijms26020442)
Supplement: Supplementary file 1 [file ijms-26-00442-s001.zip › CC_metagen_1.3 server_results/BI_1.html]

Javascript must be enabled to view this page.

magnitude
magnitudeUnassigned

results

20304

20304
36

350

350

350

350

16636

12972
80

12760

12736

292

4456

4456

4456

34

858

858

858

3942

3942
26

18

492

26

90

50

242

2670

328

314

314

210

210

210

2630

1952

1952

678

678

24

24

132

98

98

98

98

34

34

3632
22

3610

3610

3610

32

32

32

32

32

32

3282
22

712

712

712

712

712

712

1054

22

22

22

22

22

208

208

208

164

164

44

52

182

182

182

22

22

22

22

30

32

16

16

16

16

16

16

16

16

506

456

456

456

456

50

22

22

22

28

28

28

1076

882

28

28

28

854

854

854

126

126

126

126

126

24

24

24

24

24

44

44

44

44

418
