# Supplementary material for: Optimization of Compost and Peat Mixture Ratios for Production of Pepper Seedlings
Source: Int J Mol Sci. 2025 Jan 7;26(2):442. doi: 10.3390/ijms26020442 (PMC11765180; doi:10.3390/ijms26020442)
Supplement: Supplementary file 1 [file ijms-26-00442-s001.zip › CC_metagen_1.3 server_results/BI_2.html]

Javascript must be enabled to view this page.

magnitude
magnitudeUnassigned

results

86

86

86

86

44

44

16

16

16

28

28

28

42

42

42
