# Supplementary material for: Optimization of Compost and Peat Mixture Ratios for Production of Pepper Seedlings
Source: Int J Mol Sci. 2025 Jan 7;26(2):442. doi: 10.3390/ijms26020442 (PMC11765180; doi:10.3390/ijms26020442)
Supplement: Supplementary file 1 [file ijms-26-00442-s001.zip › CC_metagen_1.3 server_results/BI_3.html]

Javascript must be enabled to view this page.

magnitude
magnitudeUnassigned

results

668

668

476

394

394

394

24

338

284

284

54

54

32

82

82

82

82

192
40

108

108

108

108

108

108

44
