# Supplementary material for: Optimization of Compost and Peat Mixture Ratios for Production of Pepper Seedlings
Source: Int J Mol Sci. 2025 Jan 7;26(2):442. doi: 10.3390/ijms26020442 (PMC11765180; doi:10.3390/ijms26020442)
Supplement: Supplementary file 1 [file ijms-26-00442-s001.zip › CC_metagen_1.3 server_results/CIII_1.html]

Javascript must be enabled to view this page.

magnitude
magnitudeUnassigned

results

582964

582466
3400

302

302
262

40

6026

194

194

5832
34

5798

326660

290682
874

289808

289808
37890

792

5340

5340

5340

4640

2170

26

26

75388

932

10

10

398

52

52

10

42

10390

10390
206

72

1562

316

156

16

418

6202

1442

7916

48

48

222
200

22

22

143004
1270

108670

98

108572

32434

32434

410

24

118

78

590

590

1812

1786

26

26

26

26

1760

1760

26

26

26

26

42

42

42

42

42

34124
140

33950

33950

70

33880

34

34

34

50

50

50

50

50

18

241896
58402

98

98

48

48

50

50

115684

115096

115096

115096

1234

1234

350

350

113512
98110

13708

454

84

1156

588

588

588

588

588

27304
392

1866

1866

118

118

118

1116

1116

1116

238

238

394

394
42

352

12026

12026

10902

10226

10226

500

116

22

38

358

26

26

96
36

60

236

236

52

52

556

556

556

52

106

24

24

24

24

412

412

412
242

110

60

60

788

4968

166

130

90

28

62

62

2548
28

24

24

24

38

38

38

16

16

16

2258
28

2230

30

32

2168

88

88

60

60

36

36

2034

22

22

22

2012
1984

28

28

5374

30

30

30

4414

3732

62

62

3670
146

3524

620
552

38

30

30

32

32

14

14

16

16

748

32

32

32

654

654

654

62

62

86

86

24

24

62

62

60

60

36

36

304

304

98

98

98

206

34

172

26

146

212

938

554

554

554

40

40

268

268

76

76
42

34

26

5894
166

112

112

112

982

156

102

102

102

54

54

54

322

322

248

186

62

74

20

20

20

20

248

236

236

236

1448

1448

1448

1448
1094

354

206
188

18

18

18

18

620

408

374

34

34

142

142

142

70

70

38

32

32

32

32

32

1534

756

28

28

28

40

40

40

40

82

82

82

82

86
46

40

36

36

36

36

484

34

34

34

450

450

450

34488

478

478

24

24

454

454

454

2984
238

28

2718

284

284

284

2434

2434
326

1996

112

80

80

80

160

160

160
38

122

34

34

34

34

378

378

378

498

498
