# Supplementary material for: Optimization of Compost and Peat Mixture Ratios for Production of Pepper Seedlings
Source: Int J Mol Sci. 2025 Jan 7;26(2):442. doi: 10.3390/ijms26020442 (PMC11765180; doi:10.3390/ijms26020442)
Supplement: Supplementary file 1 [file ijms-26-00442-s001.zip › CC_metagen_1.3 server_results/CIII_2.html]

Javascript must be enabled to view this page.

magnitude
magnitudeUnassigned

results

204

204

98

98

98

98

22

42

42

42

34

106

42

42

42

42

42

42

42

42

42

42

24

18

22
