# Supplementary material for: Optimization of Compost and Peat Mixture Ratios for Production of Pepper Seedlings
Source: Int J Mol Sci. 2025 Jan 7;26(2):442. doi: 10.3390/ijms26020442 (PMC11765180; doi:10.3390/ijms26020442)
Supplement: Supplementary file 1 [file ijms-26-00442-s001.zip › CC_metagen_1.3 server_results/CIII_3.html]

Javascript must be enabled to view this page.

magnitude
magnitudeUnassigned

results

276796

275192
830

340

56

32

32

32

24

24

284

284

284
26

258

258

80

80
44

36

3512

138

138

3374
242

3132

3132

113044

58460
790

56918

1458

1458

288
100

188

1170
392

628

62

88

55396
18

34

10

18

18

18

204

3350

12

12

3338

14

3324

22316
28

22288
162

102

2942

558

190

250

36

18

78

44

1256

16

26

15016

12

1582

21232

21232

8140

8140

8140

74

64

64

64

738

16

16

224

24

164

164

164

36

36

498

498

378

378

120

14

44

44

44

44

44

526

426

258

258

258

168

44

124

124

100

100

100

40

40

40

40

40

40

58

58

58

58

58

250

62

62

62

62

62

188

188

188

188

188

38

38

38

38

38

53608
348

53260

53260

106

53144

10

20

20

20

20

20

20

141822
13724

46

46

46

46

23022
58

22872

22872

22872

256

256

512

512

22104
17790

3532

442

68

272

92

92

92

92

92

57300
544

6724

6724

32

4702

4702

52

1938
38

48

48

154

32

40

90

90

1536

1536

1144

1144

1110
24

1036

50

34

34

34

1838

1838

1838
192

1646

762

3026

120

120

92

352

640

46

46

46

484

72

38

24

24

1798

38

76

30

30

30

1654
36

1618
1558

60

18

18

42228
48

68

68

130

130

30

100

100

114

114

118

118

2588

2588

420

420

122

122

122

36580

35374
278

118

94

24

74

74

22

22

134

134

34748
34566

20

72

58

32

40

40

798
24

488
442

46

216

216

70

70

30

30

24

24

38

38

276
130

26

86

86

34

64

64

64

22

122

122

122

386

74

24

24

50

50

160

18

18

38

38

104
34

70

38

38

38

114

114

114

1446
60

1386

154

204

58

58

106

106

22

22

84

84

40

40

40

620
76

348

348

348

116

116

80
28

52

52

52

38

38

38

38

38

3418
72

32

32

32

158

158

158

158

158

454

68

68

68

68

304

304

94
52

42

210

34

176

42

40

40

40

2272

2272

2272

2272

242

2030

76

76

76

76

76

64

64

64

64

32

32

32

32

96

162

42

42

42

42

120

120

120

44312

536

340

108

108

108

164

164

164

38

38

38

38

38

30

30

30

196

16

16

44

44

44

44

136

136

136

136

172

172

172

172

172

172

1022
64

958

76

76

76

882

882
552

330

36

36

36

36

168

168

168

168
98

70

13094

13094

13094

13094

516

516

428

88

1604
