# Supplementary material for: Optimization of Compost and Peat Mixture Ratios for Production of Pepper Seedlings
Source: Int J Mol Sci. 2025 Jan 7;26(2):442. doi: 10.3390/ijms26020442 (PMC11765180; doi:10.3390/ijms26020442)
Supplement: Supplementary file 1 [file ijms-26-00442-s001.zip › CC_metagen_1.3 server_results/CII_1.html]

Javascript must be enabled to view this page.

magnitude
magnitudeUnassigned

results

6564

6564
62

44

44

44

44

44

32

32

32

32

4488

3780
580

3148

3148

688
628

60

60

28

28

28

38

38

38

44

44

44

74

74

74

2224

1796

1796

428

428

52

52

52

52

52

708

708

708

708

1916
1272

272

272

272

272

272

272

186
12

60

60

32

32

32

28

28

28

34

34

34

34

34

80

80

62

62
20

42

18

18

92

64

40

24

24

24

28

28

28

28

28

94

22

22

22

22

22

22

22
