# Supplementary material for: Optimization of Compost and Peat Mixture Ratios for Production of Pepper Seedlings
Source: Int J Mol Sci. 2025 Jan 7;26(2):442. doi: 10.3390/ijms26020442 (PMC11765180; doi:10.3390/ijms26020442)
Supplement: Supplementary file 1 [file ijms-26-00442-s001.zip › CC_metagen_1.3 server_results/CII_2.html]

Javascript must be enabled to view this page.

magnitude
magnitudeUnassigned

results

700

700

410

266

266

266

22

22

22

222

222

222

22

52

52

52

52

92

92

92

92

290
30

130

130

130

130

130

130

86

86

86

86

86

44
