# Supplementary material for: Optimization of Compost and Peat Mixture Ratios for Production of Pepper Seedlings
Source: Int J Mol Sci. 2025 Jan 7;26(2):442. doi: 10.3390/ijms26020442 (PMC11765180; doi:10.3390/ijms26020442)
Supplement: Supplementary file 1 [file ijms-26-00442-s001.zip › CC_metagen_1.3 server_results/CII_3.html]

Javascript must be enabled to view this page.

magnitude
magnitudeUnassigned

results

129106

129106
1108

7446
20

986

678

678

678

308

308

20

20

20

20

6420

342

342
86

242
214

28

14

6078

6078
828

176
126

50

20

94

4960

16

16

16

16

61000

11556
10570

848

326

326

124
26

98

202

134

18

30

20

522
34

70

156

156

156

56

56

56

136
32

84

84

20

20

70

70

70

138

56

16

40

40

40

82

82

82

82

43770

43324

56

30

26

24

24

24

43244

142

84

84

84

6356

36662

36662

422

98

98

324
34

290

34

34

256

256

24

24

24

24

24

72

72

72

2460

2460

2460

2460

2460

2460

3142

3142

3142

3142

3142

3142

13938

108

10166

22

82

82

82

82

82

82

34

5226
94

2018

2018

28

28

28

836

424

424

412

64

348

22

22

26

26

26

1106

1082

1082

24

24

44

44

44

44

44

1068

142

1522

484

78

22

56

56

358

18

18

240

20

220

42

42

58

58

48

48

48

1038

68

24

24

44

44

36

36

36

934

50

50

884

884

330

330

330

330

54

276

8

8

8

8

8

4362

84

36

36

48

48

1698

126

30

30

30

96

34

34

62

62

1408

1408

1408
1230

178

164

164

164

58

750

750

750

750

34

716

324

324

324

152

152

172

172

72

208

208

120

88

88

1148

20

98

98

98

98

98

98

342

21022

16088

6182

6182

6074

64

44

44

9126

9126

78

50

8998

720

720

720

720

36

36

24

24

4934

4934

52

52

52

4882

4882

4882

7600

7600

7600

7600

7600

7600

4760

4760

2472

2472

2232
100

20

20

2112

56

938

938

938

938

940

218

218
186

32

722

722

722

460

262

64

64

64

64
