# Supplementary material for: Optimization of Compost and Peat Mixture Ratios for Production of Pepper Seedlings
Source: Int J Mol Sci. 2025 Jan 7;26(2):442. doi: 10.3390/ijms26020442 (PMC11765180; doi:10.3390/ijms26020442)
Supplement: Supplementary file 1 [file ijms-26-00442-s001.zip › CC_metagen_1.3 server_results/CI_1.html]

Javascript must be enabled to view this page.

magnitude
magnitudeUnassigned

results

208

208

104

66

66

66

66

66

66

38

38

38

38

104

32

32

32

32

32

32

14

14

14

14

14

42

42

42

42

42

16
