# Supplementary material for: Optimization of Compost and Peat Mixture Ratios for Production of Pepper Seedlings
Source: Int J Mol Sci. 2025 Jan 7;26(2):442. doi: 10.3390/ijms26020442 (PMC11765180; doi:10.3390/ijms26020442)
Supplement: Supplementary file 1 [file ijms-26-00442-s001.zip › CC_metagen_1.3 server_results/CI_2.html]

Javascript must be enabled to view this page.

magnitude
magnitudeUnassigned

results

494

494

336

280

240

240

38

142

110

110

32

32

60

40

40

40

40

40

56

56

56

56

158
34

72

72

72

72

72

72

32

32

32

32

32

20
