# Supplementary material for: Optimization of Compost and Peat Mixture Ratios for Production of Pepper Seedlings
Source: Int J Mol Sci. 2025 Jan 7;26(2):442. doi: 10.3390/ijms26020442 (PMC11765180; doi:10.3390/ijms26020442)
Supplement: Supplementary file 1 [file ijms-26-00442-s001.zip › CC_metagen_1.3 server_results/CI_3.html]

Javascript must be enabled to view this page.

magnitude
magnitudeUnassigned

results

348

348

208

106

106

106

106

106

106

102

102

102

102

102

140

52

52

52

52

52

70

70

70

70

56

14

18
